# CerebroMatic: A Versatile Toolbox for Spline-Based MRI Template Creation

**DOI:** 10.3389/fncom.2017.00005

**Published:** 2017-02-22

**Authors:** Marko Wilke, Mekibib Altaye, Scott K. Holland, The CMIND Authorship Consortium

**Affiliations:** ^1^Department of Pediatric Neurology and Developmental Medicine, Children's Hospital and Experimental Pediatric Neuroimaging Group, Children's Hospital and Department of Neuroradiology, University of TübingenTübingen, Germany; ^2^Pediatric Neuroimaging Research Consortium, Cincinnati Children's Research Foundation and Department of Pediatrics, Division of Biostatistics and Epidemiology, University of Cincinnati College of MedicineCincinnati, OH, USA; ^3^Pediatric Neuroimaging Research Consortium, Cincinnati Children's Research Foundation and Department of Radiology, University of Cincinnati College of MedicineCincinnati, OH, USA

**Keywords:** MRI template, spline interpolation, multivariate adaptive regression splines, pediatric neuroimaging, spatial normalization

## Abstract

Brain image spatial normalization and tissue segmentation rely on prior tissue probability maps. Appropriately selecting these tissue maps becomes particularly important when investigating “unusual” populations, such as young children or elderly subjects. When creating such priors, the disadvantage of applying more deformation must be weighed against the benefit of achieving a crisper image. We have previously suggested that statistically modeling demographic variables, instead of simply averaging images, is advantageous. Both aspects (more vs. less deformation and modeling vs. averaging) were explored here. We used imaging data from 1914 subjects, aged 13 months to 75 years, and employed multivariate adaptive regression splines to model the effects of age, field strength, gender, and data quality. Within the spm/cat12 framework, we compared an affine-only with a low- and a high-dimensional warping approach. As expected, more deformation on the individual level results in lower group dissimilarity. Consequently, effects of age in particular are less apparent in the resulting tissue maps when using a more extensive deformation scheme. Using statistically-described parameters, high-quality tissue probability maps could be generated for the whole age range; they are consistently closer to a gold standard than conventionally-generated priors based on 25, 50, or 100 subjects. Distinct effects of field strength, gender, and data quality were seen. We conclude that an extensive matching for generating tissue priors may model much of the variability inherent in the dataset which is then not contained in the resulting priors. Further, the statistical description of relevant parameters (using regression splines) allows for the generation of high-quality tissue probability maps while controlling for known confounds. The resulting CerebroMatic toolbox is available for download at http://irc.cchmc.org/software/cerebromatic.php.

## Introduction

Registration of brain MR images into a common stereotactic space is an important step in MRI group analyses. Following the groundbreaking work of Talairach and Tournoux (1988), the concept of a reference brain has subsequently gained widespread support in the scientific community (Mazziotta et al., 2001; Brett et al., 2002, Evans et al., 2012). The large majority of studies published today employ a volume based registration strategy (Klein et al., 2009; Oliveira and Tavares, 2014), although surface-based (Davatzikos, 1996; Thompson et al., 1997; Lancaster et al., 1999; Frost and Goebel, 2012) or combined approaches (Fischl, 2012; Luo et al., 2014) have also been suggested.

Spatial registration is now commonly performed in the context of tissue segmentation to avoid issues of image imperfections (Bookstein, 2001; Good et al., 2001; Smith, 2002; Iglesias et al., 2011), allowing for a “per tissue class” approach to image registration (Ashburner and Friston, 2005; Ashburner, 2007). The accuracy of these approaches is improved by explicitly modeling non-brain tissue such as dura and scalp, as well as background (Ashburner, 2012; Fischmeister et al., 2013; Kazemi and Noorizadeh, 2014; Malone et al., 2015). Hence, multiclass tissue priors have replaced whole-brain T1-weighted images as the preferred target for spatial normalization, particularly in the framework of the widely-used statistical parametric mapping software (SPM, FIL, University College London).

Naturally, such a registration is only conceptually meaningful if the tissue priors are appropriate for the subject under study. Most publicly available templates in popular software solutions such as SPM, FSL, or AFNI, are based on collections of (mostly younger) healthy adults (e.g., the average age of subjects contributing to the MNI152 atlas is 25.0 ± 4.9 years, of those contributing to MNI305, 23.4 ± 4.1 years, of those contributing to the DARTEL priors in spm12, 48.6 ± 16.4 years, etc.). As a general rule, the error associated with the use of inappropriate target brains must be expected to be a function of the structural differences between the population contributing to the template and the population under study. Due to the substantial changes the human brain undergoes as part of normal human brain development (Brain Development Cooperative Group, 2012; Ziegler et al., 2012a,b), concerns have therefore been raised about the applicability of standard adult priors when studying infants, children, and adolescents, as well as elderly subjects. Consequently, several attempts have been made to provide more accurate registration targets for (in terms of algorithmic expectations) “unusual” populations (Wilke et al., 2002, 2003, 2008; Mega et al., 2005; Altaye et al., 2008; Marcus et al., 2010; Shen et al., 2012; Luo et al., 2014; Shi et al., 2014; Richards et al., 2015). While providing more accurate targets for the population in each study, the plethora of available, and not directly comparable, templates has been considered “a plague” to the field of neuroimaging, making meta-analytic approaches much more difficult (Evans et al., 2012). On the other hand, however, this disadvantage may be outweighed by the fact that accuracy, sensitivity, and specificity within a given study must be expected to be higher when using appropriate priors.

When using more extensive non-linear registration approaches, increasingly crisp template images are required to match the input images to Ashburner (2007) and Luo et al. (2014). Such high-dimensional approaches aim at increasing correspondence and at decreasing the residual group variance, representing the upper end of a continuum that has an affine-only approach on the lower end (see Figure 3B in Evans et al., 2012, for an illustration). Following more extensive deformation, however, the resulting image may not reflect the full variance inherent in the population before deformation (as much of the variance will be captured by the deformation field; Crum et al., 2003). Further, a large amount of deformation may be necessary for a given subject to achieve this end, naturally depending on the target brain (Wilke et al., 2002; Leporé et al., 2007). Also, the question whether functional organization in general, and structure-function relationship in particular, are reliable enough across individuals to make an exact correspondence of anatomical details desirable in the first place has been questioned (Brett et al., 2002; Crivello et al., 2002; Thirion et al., 2006; Eickhoff et al., 2009). Therefore, template brains were traditionally generated using a linear, affine transformation only, which will scale the image globally to match the template, but will not perform any non-linear feature matching in order to preserve the individual characteristics (e.g., Collins et al., 1994; Wilke et al., 2002, 2008). This, however, will only lead to an appropriate overlap of larger, more conserved brain structures, but not of smaller, or more variable ones. It will also tend to produce larger images due to the effect of virtual convolution (Evans et al., 1993). Consequently, a somewhat fuzzy image will result which will itself not be a good target for extensive local matching (not despite, but because, its construction was aimed to preserve the local features of the input population). On the other end of the spectrum discussed by Evans et al. (2012), an extensively-matched population will yield a very crisp resulting image (see Luo et al., 2014, or below, for an illustration). This allows for better local matching (Mazziotta et al., 2001), but at the cost of more deformation in each individual case. The cost-benefit ratio, in terms of the decrease of structural variability over subjects vs. the increase in deformation required for each subject, is not clear. Therefore, there is a need for investigating the relationship between deformation and change in variance, in particular in the context of the changes occurring in the developing brain. To this effect, three different normalization strategies (affine, low-, and high-dimensional warping) are investigated here. While tested here within the spm framework, they can be considered representative for similar approaches in other software solutions.

**Figure 1:**
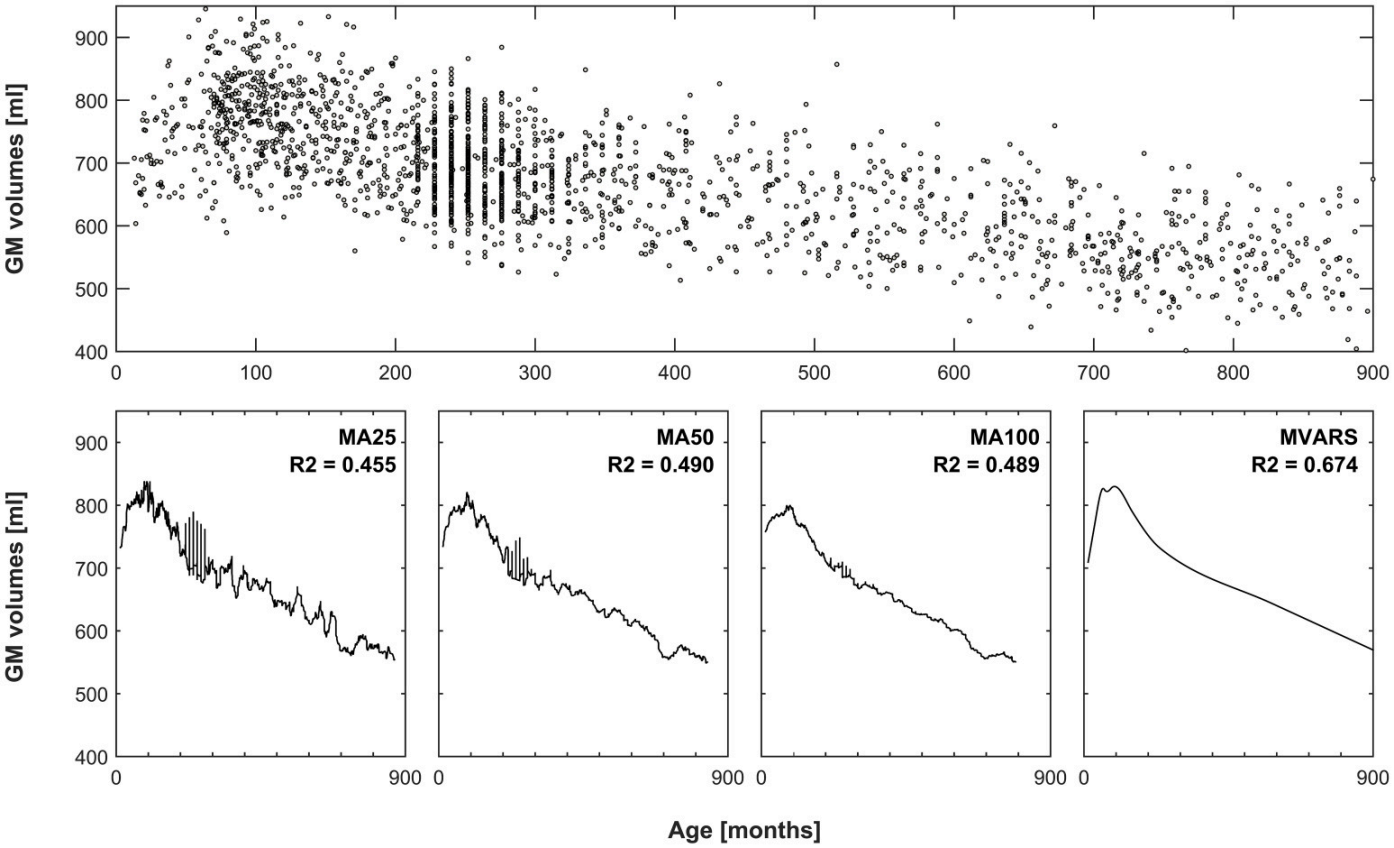
**Comparison of averaging vs. statistical modeling**. **Top panel:** global gray matter volumes versus age from all subjects, used as an exemplary dataset. **Bottom panels** 1–3: effect of using a moving average (MA) filter including 25, 50, or 100 subjects, to generate means for each timepoint. Note increasingly smoother curves with more subjects, but also increasingly pronounced “blunting” of the peak in late childhood. Left-most panel: resulting curve following multivariate adaptive regression splines (MVARS; 8 final basis functions), modeling age, gender, field strength, and data quality. The variance explained (R2) by either approach also demonstrates better modeling of the overall variance by the statistical approach.

The question of optimal template creation is important to consider for data from children in particular. As an alternative to the usual approach of averaging subject data, we previously suggested an alternative approach based on the assumption that a more appropriate representation of the salient features of a population could be achieved if the defining demographic features are described statistically (Wilke et al., 2008). We showed that age and gender were the most important contributors by far, and that their inclusion in a general linear model (GLM) allowed for the statistical description of (and thus, accounting for) their effects in a large cohort of healthy children (Wilke et al., 2008). These statistical parameters could then be used to synthetically generate a tissue probability map for a certain age, and gender, to be used as reference data in ensuing studies. The elegance of such an approach is that the resulting images are an accurate representation of only the regressors of interest (e.g., age and gender) and contain no sources of unexplained noise, making them a “cleaner” solution. However, there are limitations of the GLM, such as a vulnerability with respect to outliers and toward the edges of the dataset, and stability issues when strong correlations among the predictors are present (Petersson et al., 1999; Henson, 2006; Rorden et al., 2007; Cousineau and Chartier, 2010). Therefore, a more robust, non-parametric, yet flexible, multivariate approach may be preferable. To this effect, the multivariate adaptive regression spline approach has been suggested as an interesting alternative (Friedman, 1991). The approach can briefly be summarized as follows: to obtain the dependent responses *y*, the system that generates the data is described by

(1) $$\begin{array}{r} y=f\left(X_{1},\;\ldots ,\;X_{n}\right)=f\left(X\right)+e \end{array}$$

where *X*_1:*n*_ represent the input variables and *e* is the fitting error. Then the multivariate adaptive regression spline can be expressed as a linear combination of basis functions in the form of

(2) $$\begin{array}{l} f\left(X\right)=\theta_{0}+\overset{M}{\underset{m\;=\;1}{\sum }}\theta_{m}\;B_{m}\left(X\right) \end{array}$$

where θ_0_ is the intercept, *M* is the number of basis functions, θ_*m*_ are the basis function's coefficients (estimated by minimizing the residual sum of squares), and *B*_*m*_ is the basis function. We can use piecewise linear or cubic splines as a basis function of the form

(3) $$\begin{array}{r} {\left(x-t\right)}_{+}=\left\{\begin{array}{c} x-t,\;if\;x>t\\ 0,\;otherwise \end{array}\;\right\}\text{ and}\\ {\left(t-x\right)}_{+}=\left\{\begin{array}{c} t-x,\;if\;x<t\\ 0,\;otherwise \end{array}\right\} \end{array}$$

with a knot defined at value *t*. The modeling process uses a forward and a backward pass. The forward pass is used to try our new function products and determine which product decreases the error. The backward pass is used to avoid overfitting. During the forward pass, it uses basis function at both sides of the knot (e.g., *f*(*x*_1_, *x*_2_) = (*x*_1_ − *x*)_+_ * (*x* − *x*_2_)_+_). During the backward pass, it removes one term at a time. Due to these properties, splines can be used to describe smoothly varying trajectories by a series of continuous, piecewise, cubic polynomial functions. This framework has already been applied to neuroimaging data, to model early as well as late human brain development (Ziegler et al., 2012a; Chen et al., 2014). An exemplary application (and a comparison with conventionally-generated means) is shown in Figure 1. Their behavior is characterized by the number of basis functions (centered around knots), which makes them very flexible (Unser, 1999). To avoid overfitting, this flexibility can be penalized by placing constraints on the curvature of the resulting spline. Splines are also well-suited to model the effects of a given predictor of interest because the predicted values are linear combinations of the original response values.

This study was aimed at investigating three questions: one, when using an affine-only vs. two (more or less extensive) combined deformation approaches, in how far does the resulting decrease in variance across the population come at the cost of a higher local deformation. Two, based on demographic predictors known to explain a large portion of the variance in neuroimaging data, is it possible to model these patterns using a multivariate adaptive regression splines approach. By thus describing the pattern of normal brain development, this should allow for the creation of statistically-defined tissue priors to be used in tissue segmentation and spatial normalization. Finally, the resulting tissue priors should be compared to the standard (straight mean) approach.

## Subjects and methods

### Datasets and subjects

We compiled a large population of healthy subjects across a wide age range. For infants and children, we used data acquired as part of the *Study of Normal Brain Development* conducted by the US National Institute of Health (NIH; dataset 1) as well as from the *Cincinnati MR Imaging of Neurodevelopment study* (C-MIND; dataset 2). For adults, we used data acquired as part of the *1000 functional connectome* study (fCONN; dataset 3) as well as from the *Information eXtraction from Images* study (IXI; dataset 4). Overall, an initial sample of 2081 T1-weighted MR images was included. Due to small sample size (*n* = 20, see below) in a given 2-year age brackets, subjects with an age >900 months (75 years) were removed. Also, subjects below 1 year of age were excluded as the first year of life represents a period of rapid volumetric and signal changes (Zacharia et al., 2006; Choe et al., 2013), including the presence of unmyelinated white matter (Ashburner, 2012; Anbeek et al., 2013). For this population, dedicated segmentation and normalization routines are needed (Anbeek et al., 2013; Shi et al., 2014; Wang et al., 2014; Išgum et al., 2015). Following quality control (see below), a final sample of 1914 images was used for subsequent analyses, in the age range of 13 months to 75 years. An overview of demographic details of all subjects and datasets is provided in Table 1; more detailed information (including an overview over the data processing and analysis approach) is provided in the Supplementary Material S1.

**Table 1:** **Overview of datasets and all 1914 subjects used in this study; n, number; M, male, F, female, T, Tesla, MVR, mean voxel resolution; age and MVR are provided as means ± standard deviation [min-max]**.

	***n***	**Age [months]**	**M/F**	**3T/1.5T**	**MVR [mm ^3^]**
**Dataset 1 (NIH)**	389	130 ± 47 [20–223]	184/205	0/389	1.16 ± 0.2 [0.99–1.68]
**Dataset 2 (CMIND)**	211	100 ± 55 [13–226]	93/118	211/0	1 ± 0 [1–1]
**Dataset 3 (fCONN)**	770	333 ± 158 [96–888]	345/416	754/16	1.09 ± 0.09 [0.96–1.24]
**Dataset 4 (IXI)**	544	571 ± 188 [240–900]	242/302	366/178	1.03 ± 0.002 [1.03–1.05]
**Total**	**1914**	**333** ± **226 [13–900]**	**873/1041**	**1331/583**	**1.07** ± **0.12 [0.95–1.68]**

### Data processing: general

All data processing and analysis steps were performed in Matlab (Mathworks, Natick, MA), using functionality provided by the spm12 software package (University College London, UK) as well as customized scripts and functions. Multivariate adaptive regression spline analyses were performed using the ARESLab toolbox (Jekabsons, 2011). A pre-release version (v646) of the cat12 toolbox (Gaser, 2014) was used for data preprocessing. A 7th degree B-spline interpolation algorithm was used wherever possible during data processing in order to minimize interpolation artifacts (Unser, 1999). All other parameters were left at their default values unless specified otherwise.

### Preparation of images

In order to provide optimal starting estimates for the ensuing processing steps for images from several sources and differing field strengths, a number of pre-processing steps were initially applied to all images. First, images were roughly aligned with a target template in normalized space by coregistration (Ashburner and Friston, 1997); secondly, the origin of the image volume was set to the center of mass of the largest cluster (the head; Gaser, 2014); both steps serve to improve starting estimates. Third, as field strength is a factor when partitioning images (West et al., 2013) mainly due to the more pronounced image inhomogeneity (Marques et al., 2010), all images were preprocessed by an initial round of unified segmentation (Ashburner and Friston, 2005), to create a bias-corrected image in native space, which was then used for further processing. As we included images both from 1.5 and 3T scanners, this was done to reduce the differences in image inhomogeneity prior to the actual segmentation procedure, as discussed in Wilke et al. (2002, 2014).

### Tissue segmentation

Acknowledging the substantial structural differences between the brains of an infant and that of an elderly adult, it is immediately apparent that segmentation must not be biased by tissue priors during segmentation. To this effect, a segmentation approach without prior probabilities was used as implemented in the cat12 toolbox. In the most recent implementation (Gaser, 2014), it is an extension of the “new segment” approach (Ashburner, 2012; Malone et al., 2015), using the tissue priors only for spatial normalization (see below). The determination of the tissue classes is solely based on local features and on image intensity, based on previous work (Gaser et al., 2007); this implicitly lower reliance on prior assumptions makes this approach more appropriate when dealing with “unusual” subjects (i.e., those deviating from the assumptions). An adaptive maximum *a posteriori* approach (Rajapakse et al., 1997) is initially used to model image inhomogeneities, followed by a mixture model with three tissue classes to estimate partial volume effects (Tohka et al., 2004). Global intensity correction is achieved by a spatially-adaptive non-local means filter, which is followed by a block-wise optimized non-local means denoising (Coupe et al., 2008). Both are edge-preserving filters aimed to remove noise. Thereafter, a Markov random field model is applied to the entire image, which incorporates information about the tissue class of the neighboring voxels (Rajapakse et al., 1997). Finally, locally-adaptive segmentation is preceded by a removal of inhomogeneities within each tissue class by means of a local intensity transformation. Tissue classes are cleaned by a graph-cut skull-stripping approach as used before (Mahapatra, 2012), aimed at removing non-brain tissue such as blood vessels and meninges, followed by morphological growing/smoothing/shrinking operations to remove remaining misclassified tissue.

All voxels are classified into 6 tissue classes: gray matter (GM), white matter (WM), cerebrospinal fluid (CSF), bone, soft tissue, and background. Further, a skull-stripped, normalized version of the original T1 image was also generated. It should be noted here that the cat12-specific optimizations listed above are only applied to the first three tissue classes following an initial round of “spm-style” segmentation (Ashburner, 2012) to initiate the tissue classes. This is important to keep in mind as usually, segmentation routines expect the tissue probabilities in all 6 classes to sum to 100% (Malone et al., 2015); the potential discrepancies between the first three and the remaining tissue classes must therefore be accounted for when constructing tissue probability maps from these results. This was achieved here by adding/subtracting any remaining inconsistencies to/from the last tissue class (representing background) when generating the priors. The rationale is that the latter three tissue classes are used to explicitly model unwanted tissue/background, and can thus effectively also be used to account for discrepancies inherent in the segmentation approach as described above.

### Spatial normalization

We employed three processing strategies for spatial normalization. The first consisted of an affine-only approach, whereby images are transformed into stereotactic space by using a 12-parameter, linear transformation (Ashburner et al., 1997). There are no local or regional tissue intensity changes as all transformations are applied uniformly to the whole image volume. Consequently, the resulting overlap of local features is expected to be lowest, and group dissimilarity highest. Secondly, we used the approach implemented in the unified segmentation algorithm (Ashburner and Friston, 2005). This approach includes non-linear deformation as part of the spatial normalization scheme, but it is only aimed at achieving a regional matching of features. Consequently, the resulting group dissimilarity is expected to be lower, but at the cost of more deformation applied on the individual level. Lastly, we included a diffeomorphic registration approach aimed at extensively matching local features (DARTEL; Ashburner, 2007). Consequently, the resulting group dissimilarity is expected to be lowest, at the cost of high deformation on the individual level. For all approaches, a single DARTEL tissue prior set was used to generate tissue density maps, based on 555 healthy adult subjects with a mean age of ~48 years (Gaser, 2014).

### Quality control

For quality control, all images were initially loaded into a movie loop to identify overt processing failure. Thereafter, they were inspected individually twice by a single experienced rater (MW) blinded to the epidemiological information. Gray/white differentiation in the difficult-to-segment regions of the basal ganglia and thalamus (Nugent et al., 2013) and the cerebellum (Price et al., 2014) served as indicators of successful processing. Segmentation accuracy was therefore judged in an axial plane at the level of the basal ganglia in a first round, and in a coronal plane at the level of the cerebellum in a second round. Images with insufficient tissue differentiation in either round, or evidence of too lenient or too aggressive non-brain tissue removal, were excluded.

In addition to visual quality control, further measures of data quality are routinely calculated by the cat12 toolbox. These are the mean voxel resolution of the input image as well as indicators of contrast- and inhomogeneity-to-noise ratios (as determined from the white matter class; Gaser, 2014). These indicators were combined into a single measure of data quality by summing their *z*-transformed values. They were then included in ensuing analyses as an indicator of data quality.

### Definition of defaults

Due to the constraints placed on splines, they can be made to be very robust toward outliers, or data sparsity. However, it is still important to ensure that an appropriate number of subjects is available for a given age range. As this number is not ultimately known (and must be expected to vary with age; Wilke and Holland, 2003; Oishi et al., 2013), it was decided to use previously determined minimal sample sizes (Wilke et al., 2014) as a guideline so that a minimum number of 20 subjects per 2-year age bracket was used. Any 2-year bracket not satisfying these criteria was deemed inappropriately covered, and was removed from analyses. Further, in order to stabilize the fitting particularly toward the margins of the dataset, it was defined that a minimum of 20 subjects was required between knots, and a minimum of 10 subjects was required between a knot and the beginning/end of the dataset. The penalty value, constraining the curvature of the splines, was derived from the globals of each dataset under examination with a *k*-fold cross-validation, with k set to 5. Finally, splines can be fitted using an iterative piecewise linear or cubic modeling approach (Friedman, 1991). We opted to use a cubic modeling for our analyses to better preserve the smoothly varying pattern of changes with age. All of these settings were adapted to yield best results for the dataset at hand, but may (have to) be adapted for other datasets of a different size and/or composition.

Within the spline fitting process, an initial (forward) selection phase is used, typically resulting in an overfitted model. This is therefore followed by a second (backward) deletion phase that is used to prune the model by iteratively removing pairs of basis functions in order to identify the best combination of model simplicity and model fit. For the final fit, a total of 8 basis functions was assumed to be sufficient; for the pruning phase, a maximum of 40 initial basis functions (recommended: 5 times the number of final functions; Jekabsons, 2011) was therefore set. In order to restrict processing to only those regions relevant for the tissue class under consideration, only voxels with an average tissue probability exceeding 10% were included.

### Demographic predictors

Previously, we demonstrated that age and gender explain the largest amount of variance during human brain development in childhood and adolescence (Wilke et al., 2008), and these factors are also expected to be important for the rest of the lifespan (Mega et al., 2005; Marcus et al., 2010; Jernigan et al., 2011; Ziegler et al., 2012a,b; Ruigrok et al., 2014). Further, field strength may be a factor even when segmenting images (West et al., 2013; Wilke et al., 2014). These three predictors (age [in months], gender, and field strength) were therefore provided as inputs to the spline fitting algorithm (in addition to the data quality indicator, see above).

### Indicators of deformation and dissimilarity

As mentioned above, the amount of deformation on the individual level must be expected to be inversely related to the variance on the group level. The following parameters were therefore assessed: first, the affine scaling occurring during the spatial normalization scheme was determined. As the initial step of spatial normalization, this is identical over all spatial normalization approaches investigated here and was determined by calculating the determinant of the affine transformation matrix. We could previously show that this overall scaling does not correlate with age in the range between 6 and 18 years (Wilke et al., 2002), but including younger children as well as significantly older adults could change that assessment for the wider age range included here. As an indicator of the amount of deformation occurring during non-linear spatial normalization, we then calculated the sum of (absolute) Jacobian determinants over all voxels. Briefly, the Jacobian Determinant reflects the relative volume changes a voxel undergoes during spatial normalization (Good et al., 2001). The impact of the affine transformation was already calculated above and was thus removed here; these values therefore only reflect the overall amount of volume change occurring as a function of non-linear deformation. For this calculation, only voxels within the brain (as determined by the accompanying, skull-stripped T1 image) were considered.

As a measure of group dissimilarity of the original images, a mean image was calculated for each tissue class and subtracted from each individual image. The resulting sum of absolute voxelwise differences serves as an indicator of divergence-from-the-mean, for each spatial normalization approach (affine, unified segmentation, and DARTEL). This measure was chosen here as more advanced image similarity measures (such as the structural similarity index; Wang et al., 2004) would be unduly biased by the large amount of irrelevant background voxels. Following statistical analyses for each approach, synthetic tissue maps (one for each month of age) were generated and the procedure was repeated, yielding an indicator of dissimilarity in the statistically-described samples as well.

### Tissue homogeneity and explained variance

Voxels within a given brain region, in particular within a given tissue class, cannot be expected to be independent observations. Hence, it is not meaningful to assume spatial independence when fitting the spline models. We explored several options to enforce such homogeneity. Option 1 represents the most conservative approach where an initial model fit was performed on the global signal intensity values for each class, and the individual voxel results were restricted to follow the thus-derived global pattern. Option 2 is a somewhat less strict approach, in that the initial model fit to the globals was not pruned; this overfitted model was then applied to each voxel (where it is pruned), allowing more flexibility for these individual results. Option 3 represents an even less conservative approach, in that only some aspects of the global model are used as inputs for the individual models (namely the number of basis functions and the minimum distance between knots). Finally, option 4 represents the most liberal approach, in that no initial global analysis was performed and each voxel was fitted independently.

These four options must be expected to yield different levels of homogeneity in the resulting tissue classes. To investigate this, we implemented an approach similar to a Markov random field (Rajapakse et al., 1997), in that every voxel with a probability exceeding 10% in the resulting tissue probability maps was identified, and its value was then subtracted from all surrounding (26) voxels; the mean absolute deviation (in % intensity) was then analyzed across all voxels. In a fully homogeneous setting, this difference would be 0; a larger deviation over the whole volume therefore reflects a less homogeneous image. It should be noted that this measure will also be influenced by a different surface-to-volume ratio of a given tissue class, it is consequently not directly comparable across the different approaches. All results were therefore related to the inhomogeneity found in the respective tissue class in the first option.

The variance explained by the spline fit was also determined, again for each voxel and each tissue class, by calculating *r*^2^. This parameter directly reflects the proportion of variance that was explained by the model, and can serve as a measure of the goodness of fit. For comparison purposes, this value was also determined by fitting a GLM to each voxel, assessing the amount of variance explained by the same set of predictors (age, gender, field strength, and quality); here, age was also submitted in a quadratic and a cubed extension to account for non-linear effects (Wilke et al., 2008). Again, all results were related to the variance explained by the respective first option (which consequently is set to 1).

### Generation of tissue priors

As introduced above and shown before (Wilke et al., 2008), one of the advantages of the statistical description of regressors of interest is the ability to generate synthetic maps based on new predictors. Naturally, these will have to be within the range of the original predictors (age, gender, and field strength). The image quality indicator, if used in the initial analysis, is automatically set to the best-available value during prior generation. In analogy to our previous approach (Wilke et al., 2008), the algorithm initially generates one matching set of tissue classes per input item and will average results in the end (“matched pairs”).

In order to further enforce tissue homogeneity during the generation of the tissue priors, we performed a simple 3D median filtering of the resulting maps (Garcia, 2015). This was done to avoid the loss of spatial specificity inherent in Gaussian smoothing.

To provide an illustration of the resulting final images, synthetic GM, and WM tissue priors were generated for 2, 12, 32, 52, and 72 year old subjects, for each data processing approach. Further, to illustrate the covariate effects, tissue maps were generated for the mean age (333 months) for male and female gender, for high and low field strength, and for best and worst data quality settings. These maps were then subtracted from each other, and tissue probability differences exceeding 5% were visualized as colored overlays.

### Comparison of cerebromatic priors with standard approach

A normally-distributed and sufficiently-sized population will be best reflected in its own mean; the overall mean of a large population can therefore be considered the gold standard. Of note, due to the dynamic changes occurring in childhood, this assumption would not be met in a pediatric population: we therefore defined a large and homogeneous population of adults from 18 to 48 years of age. In this subgroup, a total of 943 subjects was available, mean age 26.59 ± 7.98 years, 443 males, with 98 datasets from 1.5T scanners. The assumption of normality was assessed in each voxel for GM and for WM. Three scenarios were defined, with group sizes of 25, 50, or 100 subjects (cf. Figure 1) which were randomly picked from the population. Their GM and WM maps were averaged, to reflect the standard way of custom template creation. Based on the demographic predictors of these subjects, matching CerebroMatic priors were also generated. This procedure was repeated 100 times. All of these maps were then compared to the gold standard (the overall group mean), again using the sum of absolute voxelwise differences as described above.

In a second set of simulations, the effect of decreasing the size of the control population was assessed. Again, 25 subjects were randomly picked from the above-defined subpopulation (*n* = 943), their predictors were used to generate matching CerebroMatic priors, and they were again compared to the overall group mean as detailed above. This procedure was repeated 10 times. Then, the size of the subpopulation was iteratively decreased in steps of 25 subjects, and the above procedure was repeated, until a minimum number of 43 subjects was reached.

### Statistics

Normality in the data was assessed using the Kolmogorov–Smirnov-Liliefors-Test. If the assumption of normality was met, a Student's *t*-test was used to assess significant differences between group means for the examination of continuous variables. For data that was not normally-distributed, statistical comparisons were likewise done using the non-parametrical Mann–Whitney-*U*-Test. Significance was assumed at *p* ≤ 0.05, Bonferroni-corrected for multiple comparisons where appropriate.

## Results

### Deformation and dissimilarity

**Figure 2:**
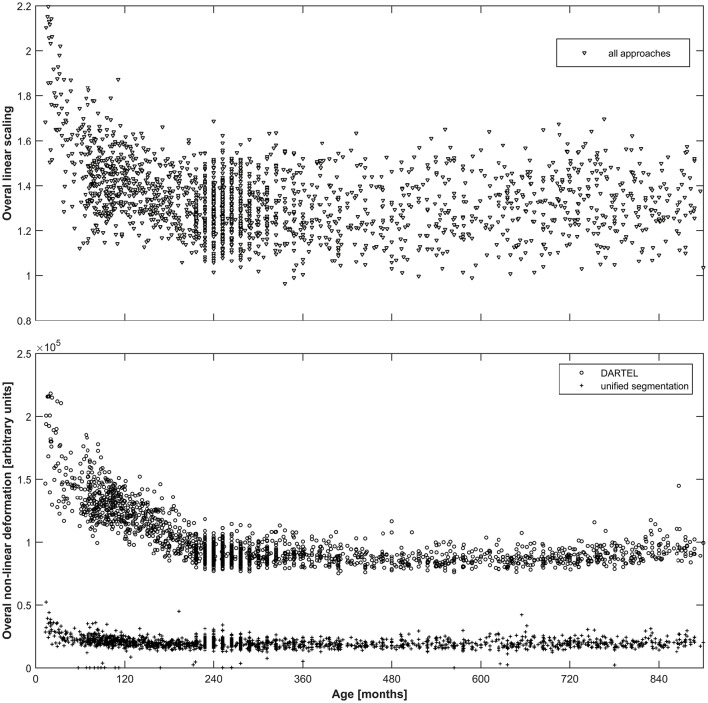
**Top panel:** Illustration of the overall linear scaling applied to the input images; note substantial increase in childhood and adolescence as well as wide variability even in adulthood. Note that this value is identical across all three approaches. **Bottom panel:** Illustration of the overall non-linear deformation applied to the input images for the DARTEL (**o**) as well as the unified segmentation (+) approach, in arbitrary units; note about five-fold higher deformation occurring in the DARTEL approach and stronger modeling of age-related effects. Note that no non-linear deformation occurs in the affine approach.

As can be seen from Figure 2, both the overall linear (top panel) as well as the overall non-linear (bottom panel) scaling exhibit substantial variation over the age span included. For the linear scaling (which is identical for all normalization approaches), the average scaling in adulthood is about 1.4, increasing substantially to values exceeding 2 in the younger infants. Non-linear deformation only occurs in the unified segmentation and the DARTEL approach and is substantially higher in the latter, with again a clear increase in younger subjects.

**Figure 3:**
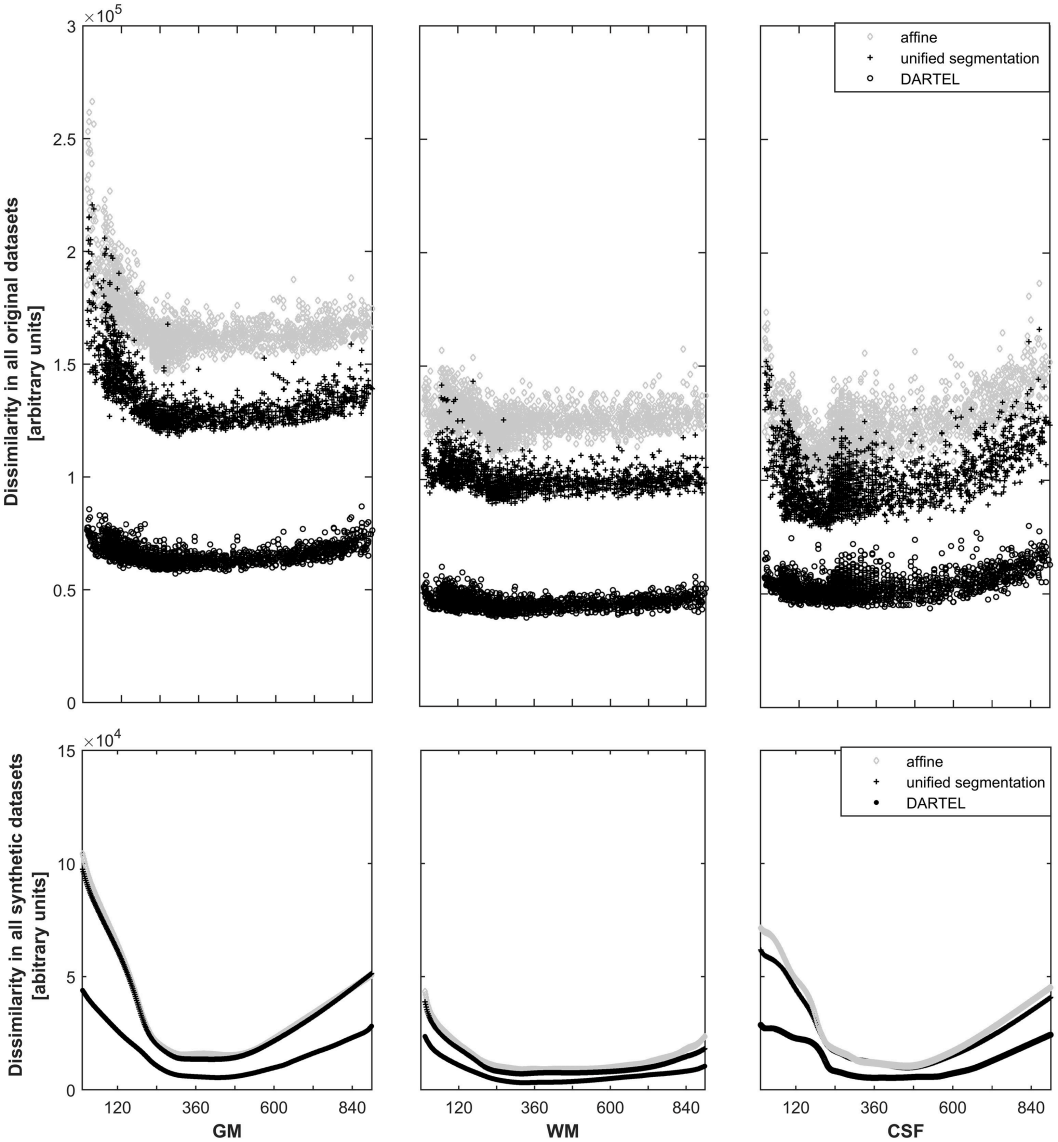
**Top panels:** Illustration of the dissimilarity inherent in all datasets in gray matter **(left panels)**, white matter **(middle panels)**, and cerebrospinal fluid **(right panels)**. Note substantially higher dissimilarity, and more obvious effects of age, in the affine (**o**) and unified segmentation (+) datasets; both effects are substantially lower in the DARTEL class (**o**). **Bottom panels**: Illustration of the dissimilarity inherent in all synthetic datasets (note identical scaling). Again, age effects are much less pronounced in the DARTEL approach as compared to the unified segmentation and affine approach.

For each approach, the dissimilarity shows the expected pattern for the primary tissue classes (see Figure 3, top panels). The highest dissimilarity following spatial normalization is present in the affine-only group, for each tissue class; the unified segmentation approach shows a lower dissimilarity, and dissimilarity in the DARTEL approach is substantially lower still. When assessing this parameter in the corresponding, synthetically-generated tissue maps (Figure 3, lower panels), it is immediately apparent that the dissimilarity itself as well as the variance is much lower (note identical scaling when compared with the upper panels). Consequently, the effect of age is still obvious in the affine and unified segmentation approach, but again much less pronounced in the DARTEL approach.

### Homogeneity and explained variance

Table 2 illustrates the effect of the four analysis options on the homogeneity in the resulting primary tissue classes. The most conservative option 1 (using a fully pre-specified model for all voxels) yields tissue maps with the lowest inhomogeneity, while the least conservative option 4 (no constraints) leads to the most inhomogeneous maps. This pattern is consistent across all processing approaches for GM and WM, with only few exceptions for CSF.

**Table 2:** **Tissue homogeneity (arbitrary units) for all approaches and processing options 1–4 for the three primary tissue classes gray matter (GM), white matter (WM), and cerebrospinal fluid (CSF)**.

	**Option 1**			**Option 2**			**Option 3**			**Option 4**		
	**GM**	**WM**	**CSF**	**GM**	**WM**	**CSF**	**GM**	**WM**	**CSF**	**GM**	**WM**	**CSF**
Affine	1.00 ± 0.60	1.00 ± 0.67	1.00 ± 0.54	1.05 ± 0.61	1.05 ± 0.68	1.01 ± 0.54	1.16 ± 0.66	1.18 ± 0.76	1.10 ± 0.59	1.17 ± 0.64	1.20 ± 0.75	1.14 ± 0.59
Unified segmentation	1.00 ± 0.68	1.00 ± 0.75	1.00 ± 0.55	1.07 ± 0.66	1.04 ± 0.76	0.98 ± 0.52	1.12 ± 0.67	1.11 ± 0.78	1.04 ± 0.53	1.12 ± 0.66	1.12 ± 0.77	1.05 ± 0.53
DARTEL	1.00 ± 0.47	1.00 ± 0.71	1.00 ± 0.41	1.01 ± 0.46	1.01 ± 0.70	0.97 ± 0.39	1.02 ± 0.46	1.02 ± 0.70	0.98 ± 0.38	1.02 ± 0.46	1.02 ± 0.70	0.99 ± 0.38

*For all classes and all approaches, the difference to option 1 is significant (all p < 0.05, Mann–Whitney-U-test, Bonferroni-corrected for multiple comparisons). All results are shown in relation to option 1 in each approach (which is set to 1); see text for details*.

Table 3 illustrates the effect of the four analysis options on the explained variance in the resulting primary tissue classes. While the absolute differences across approaches may seem minor, both across approaches and across tissue classes, the most conservative approach explains significantly less variance in all comparisons. Of note, the conventional GLM approach outperforms the spline-based modeling only when using the most conservative approach. Despite using more regressors, the GLM explains significantly less variance than the spline-based approach (option 2) in targeted *post-hoc* comparisons.

**Table 3:** **Explained variance (***r***^**2**^) for all approaches and processing options 1–4 for the three primary tissue classes gray matter (GM), white matter (WM), and cerebrospinal fluid (CSF)**.

	**Option 1**			**Option 2**			**Option 3**			**Option 4**			**GLM**		
	**GM**	**WM**	**CSF**	**GM**	**WM**	**CSF**	**GM**	**WM**	**CSF**	**GM**	**WM**	**CSF**	**GM**	**WM**	**CSF**
Affine	1.00 ± 1.12	1.00 ± 1.57	1.00 ± 0.79	1.05 ± 1.16	1.18 ± 1.71	1.09 ± 0.80	1.08 ± 1.16	1.26 ± 1.73	1.11 ± 0.80	1.08 ± 1.16	1.24 ± 1.71	1.12 ± 0.80	1.02 ± 1.10	1.10 ± 0.54	1.04 ± 0.77
Unified segmentation	1.00 ± 1.08	1.00 ± 1.57	1.00 ± 0.66	1.06 ± 1.12	1.13 ± 1.69	1.05 ± 0.67	1.07 ± 1.12	1.24 ± 1.71	1.07 ± 0.67	1.07 ± 1.11	1.21 ± 1.69	1.07 ± 0.67	1.01 ± 1.06	1.06 ± 1.51	1.00 ± 0.65
DARTEL	1.00 ± 0.90	1.00 ± 1.34	1.00 ± 0.75	1.07 ± 0.92	1.29 ± 1.58	1.08 ± 0.75	1.10 ± 0.92	1.40 ± 1.60	1.11 ± 0.75	1.09 ± 0.92	1.36 ± 1.59	1.09 ± 0.75	1.01 ± 0.88	1.15 ± 1.37	0.99 ± 0.71

*For all classes and all approaches, the difference to option 1 is significant (all p < 0.05, Mann–Whitney-U-test, Bonferroni-corrected for multiple comparisons). In targeted comparisons, option 2 also explains significantly more variance than the GLM approach (all p < 0.05, Mann–Whitney-U-test, Bonferroni-corrected for multiple comparisons). All results related to option 1 in each approach (which is set to 1); see text for details*.

### Resulting tissue priors

**Figure 4:**
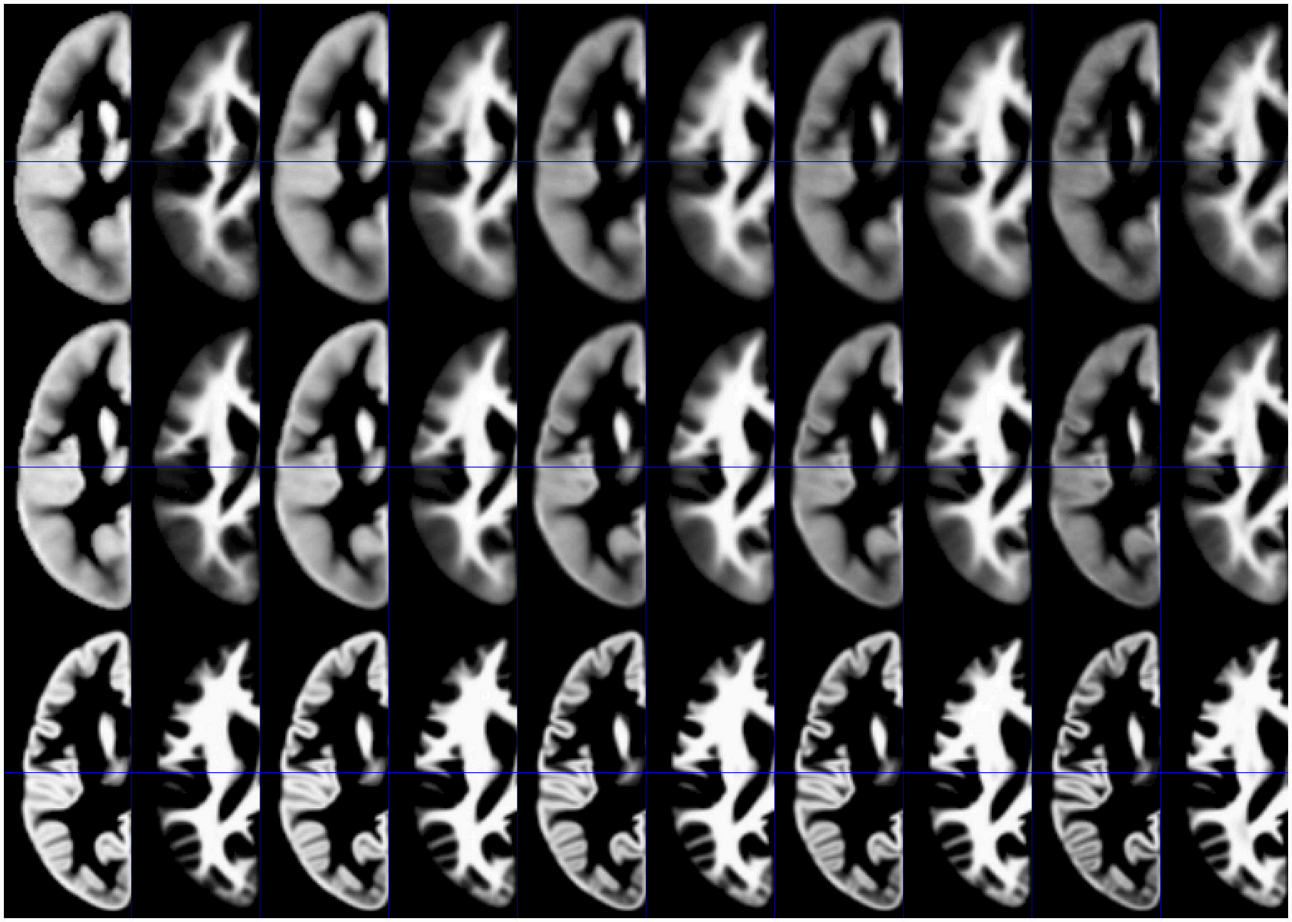
**Illustration of synthetic tissue maps for gray matter and white matter, generated for 2, 12, 32, 52, and 72 year-old subjects, using option 2**. Note more apparent effects of age in the affine **(top panels)** and the unified segmentation **(middle panels)** when compared with the DARTEL approach **(bottom panels)**.

Synthetic tissue priors for 2, 12, 32, 52, and 72 year old subjects, for GM and WM, and for each processing approach, are shown in Figure 4.

**Figure 5:**
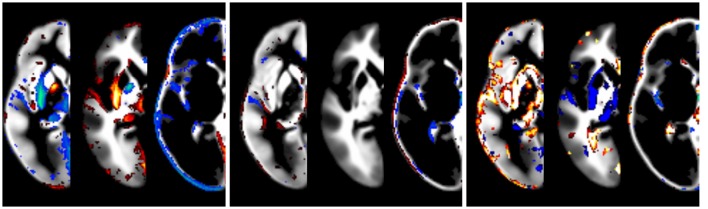
**Illustration of the effect of covariates, overlaid over synthetic tissue maps for gray matter, white matter, and cerebrospinal fluid for a subject of mean age in this population (333 months)**. Shown are the effects of statistically modeling field strength (**left panel**, 1.5 vs. 3T), gender (**middle panel**, male vs. female), and data quality (**right panel**, best vs. worst setting). Only tissue probability differences exceeding 5% are shown (in red, below −5%, or blue, above 5%).

The effect of modeling the covariates is illustrated in Figure 5. While the impact of field strength is systematic and robust, the effect of gender is lower. The effect of data quality is also strong, but less systematic. Note that the effect is greatest at the tissue boundaries, emphasizing the importance of modeling these confounds in order to achieve crisp segmentation at tissue interfaces.

### Comparison with standard approach

**Figure 6:**
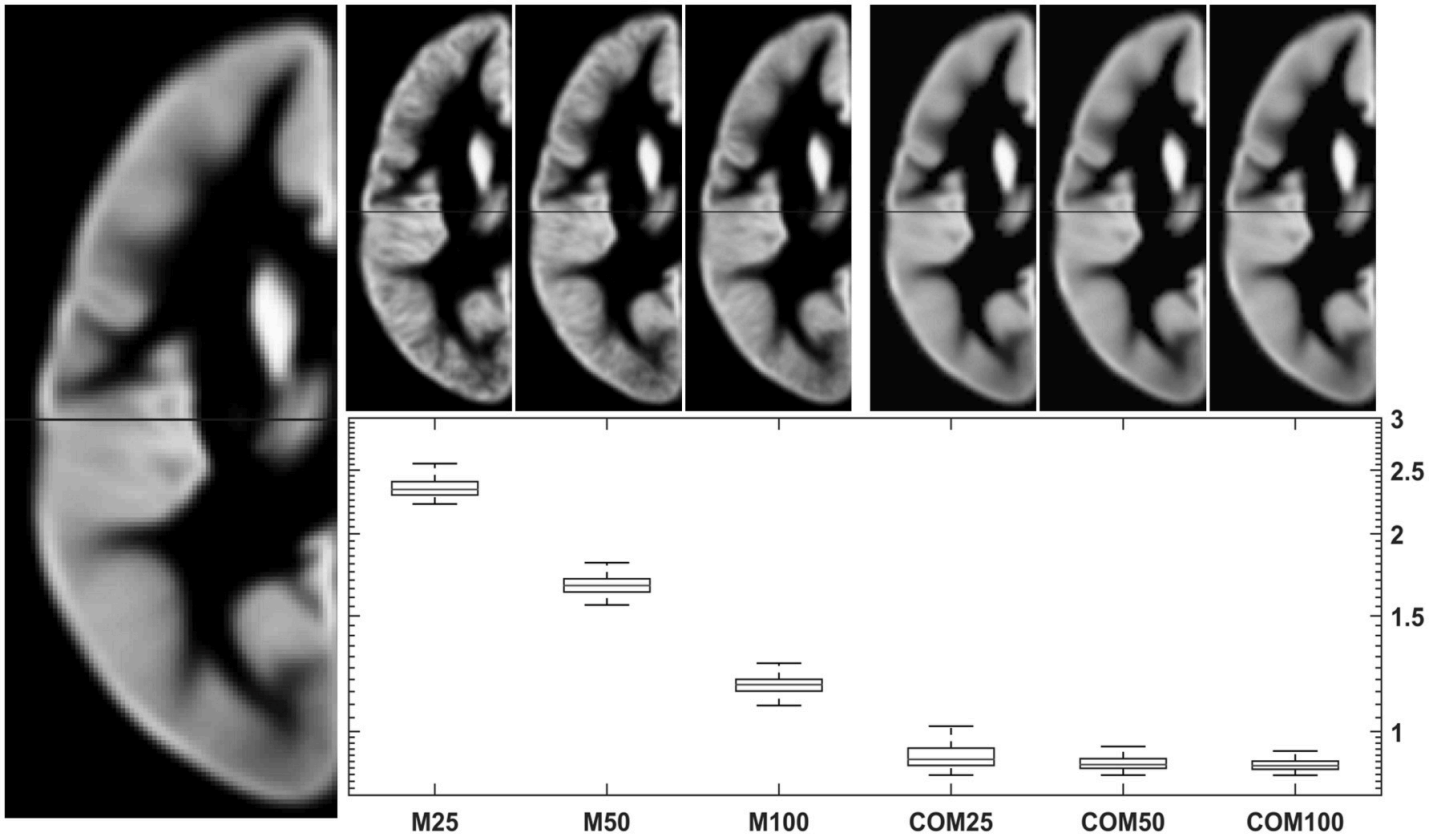
**Comparison of conventional (left upper panels)** with new **(right upper panels)** approach to GM template creation. Tissue maps were created 100 times from 25, 50, and 100 subjects using the mean (M25/50/100) or the CerebroMatic (COM25/50/100) approach and compared with the gold standard (population mean, *n* = 943; left large image). Shown in the boxplot insert are the sums of absolute voxelwise disagreements. Note increasingly lower disagreement with more subjects in the conventional approach and consistently lower disagreement for all CerebroMatic approaches.

**Figure 7:**
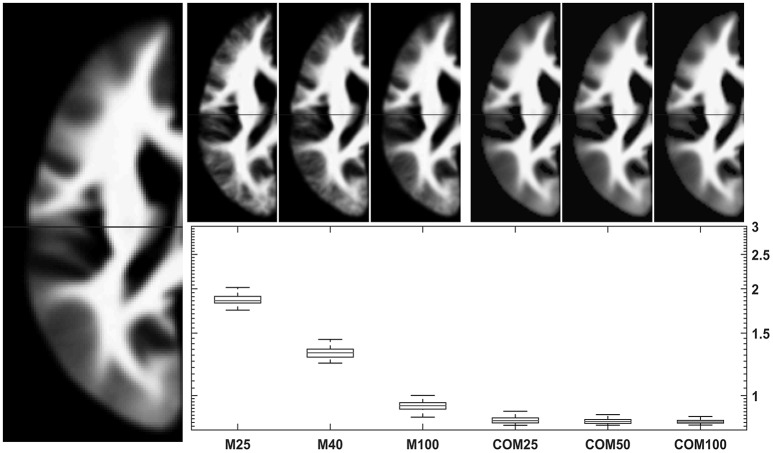
**Comparison of conventional (left upper panels)** with new **(right upper panels)** approach to WM template creation. Tissue maps were created 100 times from 25, 50, and 100 subjects using the mean (M25/50/100) or the CerebroMatic (COM25/50/100) approach and compared with the gold standard (population mean, *n* = 943; left large image). Shown in the boxplot insert are the sums of absolute voxelwise disagreements. Note increasingly lower disagreement with more subjects in the conventional approach and consistently lower disagreement for all CerebroMatic approaches.

Testing the 18-48-year-old subgroup (with *n* = 943) for normality showed that 99.94% of GM voxels and 99.93% of WM voxels in this population followed a normal distribution. Results for comparing group averages of 25, 50, or 100 subjects (and their matching CerebroMatic priors) with the overall population mean are shown in Figure 6 for GM and in Figure 7 for white matter. Note substantial effect of increasing group size on the visual quality of the conventional priors (and consequently, decreasing difference to the overall group mean), as well as consistently better agreement of the CerebroMatic prior with the gold standard, for both gray and white matter.

**Figure 8:**
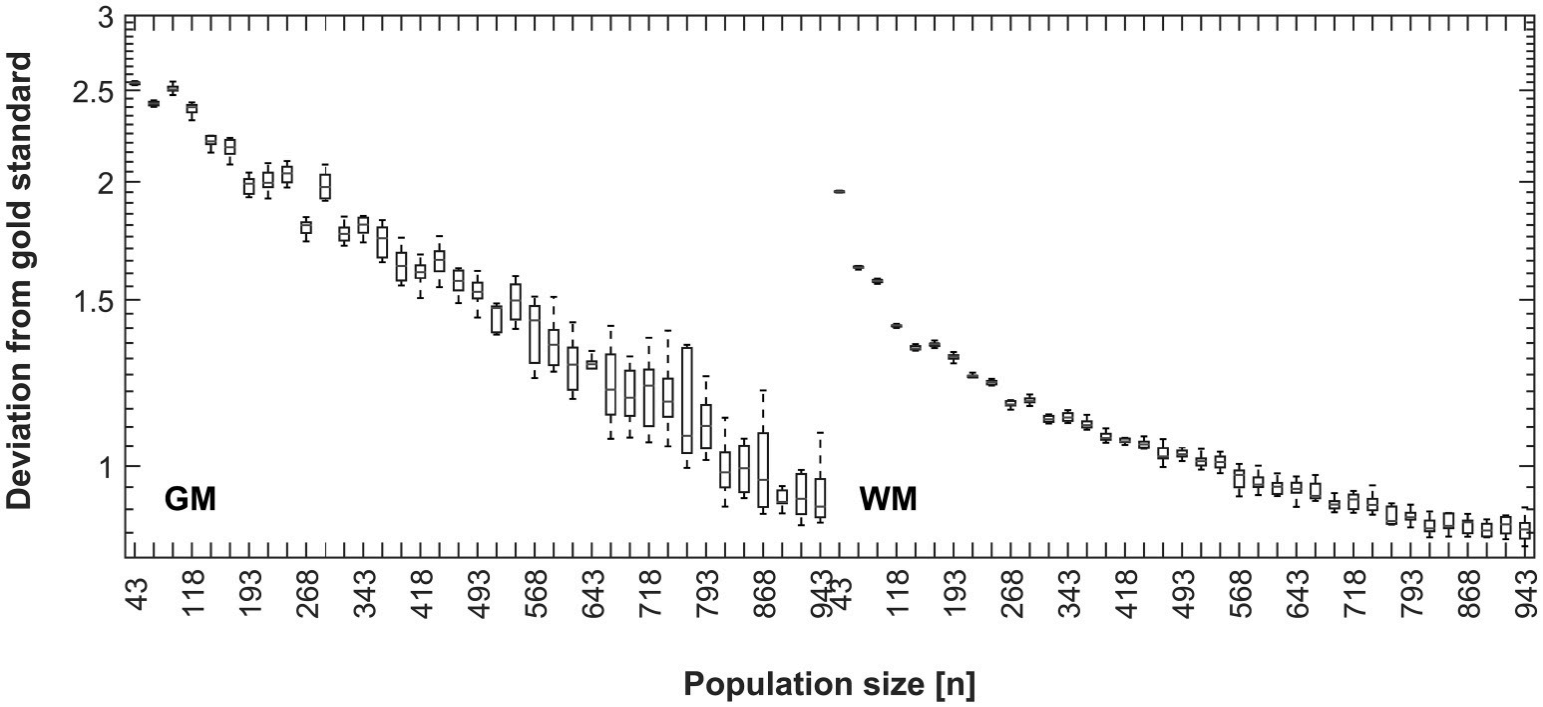
**Disagreement between CerebroMatic priors (based on 25 random subjects, 10 simulations; GM, left, WM, right) and the respective population mean, as a function of increasing population size**. Note consistently lower disagreement of the CerebroMatic priors with the mean of a larger control population.

Results for decreasing the size of the control population are shown in Figure 8. There is a decreasing mean error in the correspondence of the synthetic tissue priors as the population size increases, indicating a higher correspondence of the overall population mean with the synthetic priors when the overall population is larger. This trend continues until the maximum (*n* = 943) for GM, but shows a tendency to level off for WM at around 800 subjects.

## Discussion

This work was aimed to first, explore the trade-off between the decrease in variance achieved on the group level on the one hand and the amount of tissue deformation required on the other hand. Secondly, we also investigated the applicability of multivariate adaptive regression splines to generate new, statistically defined tissue class prior probability maps over the full lifespan from 1 to 75 years of age. Finally, we wanted to compare our new with the conventional approach to template creation. This algorithm has been compiled into a toolbox (termed “CerebroMatic”) for use within the spm software environment and is made freely available.

### Affine and non-affine deformation as a function of age and approach

The aim of the linear/affine part of the spatial transformation is to achieve a global similarity between images, such that their overall size and orientation is comparable (Ashburner et al., 1997). We previously showed that overall scaling does not correlate with age in children older than 6 years of age (Wilke et al., 2002). The current dataset affirms this previous finding; however, the overall scaling in younger children clearly does change with age, from a mean of about 1.4 in adults (very similar to previous analyses; Ashburner et al., 1997) to values exceeding 2 in the younger children and infants (Figure 2, upper panel). This is of course well in line with the substantial growth occurring in the developing human brain in the first years of life which then reaches almost adult size by age 6 (Huttenlocher, 1979; Brain Development Cooperative Group, 2012). This is of practical relevance in so far as these smaller brains deviate from the expected range and will therefore require stronger regularization to match adult templates (Ashburner et al., 1997). However, determination of the affine transformation is performed in an iterative fashion in the cat12 toolbox (Gaser, 2014), and this effect can also be accounted for by using a different affine regularization scheme (e.g., “none”). Given the dramatic volume changes in early infancy, the effect is likely even more pronounced in the first year of life, again suggesting that specialized processing approaches should be used for babies (Anbeek et al., 2013; Shi et al., 2014; Wang et al., 2014; Išgum et al., 2015).

The overall amount of non-linear deformation is substantially different between the approaches, with about 5-times more deformation occurring in the DARTEL-approach than in the unified segmentation approach on average (by definition, this value is 0 in the affine-only approach). Notably, the changes with age are much more pronounced in the DARTEL-approach, with a substantially higher deformation being exerted on the brains of children and adolescents (Figure 2, lower panel). Very old individuals (>75 years) were removed from the dataset to avoid data scarcity, but a trend to more deformation at the upper age range is also apparent. This reflects the expected effect that more “unusual” brains will be more extensively deformed in the DARTEL approach to match the template (which in this case was based on adults with a mean age of ~48 years). With less deformation being exerted in the unified segmentation approach, the interaction with age is not as apparent here.

### Group dissimilarity as a function of approach

The higher deformation exerted during the more extensive spatial normalization approaches (Figure 2) leads to a substantial decrease in resulting group dissimilarity (Figure 3; upper panels). The affine approach shows the strongest dissimilarity, followed by the unified segmentation and the DARTEL approach; this pattern is consistent across the three primary tissue classes (GM, WM, and CSF). Of note, the clear effect of age, particularly in the first two decades, is still very apparent in the first two groups, but more blunted in the DARTEL approach. This, as expected (Crum et al., 2003), demonstrates that the residual variability observable in this latter approach is substantially decreased due to the more extensive matching occurring during spatial normalization; group variability in this approach is therefore to a large extent contained in the deformation fields, not in the resulting images. Consequently, the resulting images will look much more similar than they originally were (cf. Figure 4, below). In structural MRI studies, this bias of potentially different deformation between groups is often accounted for by incorporating a measure of local volume change into each voxel; this process (usually achieved by modulation with the Jacobian determinant of the deformation matrix; Good et al., 2001) allows comparing tissue volumes, instead of tissue density, and thus partially alleviates this confound. Notably, this confound also exists for functional MRI studies comparing systematically different groups (Dorn et al., 2014), the minimization of deformation is therefore also of relevance in the fMRI setting. However, tissue priors used for spatial registration and tissue segmentation must reflect tissue density, and thus, probabilities to find this tissue class in a given voxel; hence the incorporation of volume changes into the tissue is not possible.

When assessing the effect of age as detected by the adaptive splines approach (Figure 3, lower panels), the overall pattern of age-dependency is still very apparent in the affine-only and in the unified segmentation approach, but is again substantially lower in the DARTEL approach. This demonstrates that the variance ascribable to age shown to be present in the less-deformed datasets (see Figure 3, upper panels) is appropriately captured and described by the statistical analysis using regression splines. Consequently, the generated template of a 2-year-old will be more different from that of a 25-year-old when using the affine-only or the unified-segmentation approach, while they will appear more similar when using the more extensive DARTEL-approach (see below and Figure 4). Considering these arguments as well as the results presented in Figures 2, 3, we believe that using the unified segmentation approach for data processing is the best compromise between reducing variance and enforcing anatomical overlap, in this setting.

One of the motivations for this study was our conviction that the final template should be a reflection of the meaningful biological variance inherent in the original population, and that a “non-standard” population (such as infants, or older adults) should not be processed using a standard template (Wilke et al., 2002, 2003, 2008; Mega et al., 2005; Altaye et al., 2008; Marcus et al., 2010; Shen et al., 2012; Luo et al., 2014; Shi et al., 2014; Richards et al., 2015). At the same time, newer algorithms require crisper templates to be used in order to use a more extensive matching to achieve better overlap of structures (and thus, presumably increase the likelihood to detect similarities and differences between groups; Mazziotta et al., 2001; Luo et al., 2014). The sample investigated here, with a very wide age range, admittedly is an extreme example of inhomogeneity, but exactly this inhomogeneity allows for some clear conclusions to be drawn from the results presented. One, the natural sample-inherent inhomogeneity is not preserved when using extensive matching schemes such as DARTEL. Indeed, this is not surprising at all as it is the very aim of such approaches to reduce this variance in the resulting images (Ashburner, 2007), with much of it instead being captured in the resulting deformation fields (Crum et al., 2004). The question then is whether it is appropriate to use such a scheme to generate a template. In a setting of conventional template creation, for example by using straight averages (Wilke et al., 2002, 2003), this may be more desirable as otherwise the resulting images will be very fuzzy, and anatomical features may be ill-described (particularly in smaller groups; Wilke et al., 2008). On the other hand, the presence of the group-inherent variability has been considered an important factor when creating a template, and has led to the suggestion to use registrations to multiple atlases to detect the full range of abnormalities (Min et al., 2014). In a setting such as the one suggested here, where parameters of interest are statistically described, it also seems counterproductive as the very basis for this fit (e.g., age-related variance) is already removed from the group (see Figure 3). We therefore conclude that a more extensive local matching approach is not helpful in this specific setting investigated here. However, the answer to this question cannot be generalized as a researcher investigating healthy adults may well decide to use the much more crisp DARTEL-based synthetic priors (cf. Figure 4) as the lack of captured age-related variance toward the end of the dataset is irrelevant in this context.

When assessing the performance of the multivariate adaptive regression splines approach to model the data, we were interested in the amount of inhomogeneity present in the resulting (synthetic) datasets, as well as in the variance explained by it. With regard to inhomogeneity, several points need to be considered. From a biological plausibility point of view, it is well-established that there is a wide variability in the pattern of healthy brain development and ageing, with regional patterns not necessarily following the global one (Jernigan et al., 2011; Brain Development Cooperative Group, 2012; Ziegler et al., 2012a,b); a rigid regularization of all models/voxels in a given tissue class may therefore be too strict. On the other hand, neighboring models/voxels must be expected to follow a similar pattern; an independent modeling of these trends may therefore be too lenient. We therefore opted to investigate 4 different scenarios to provide the algorithm with prior information when modeling a single voxel, from providing a completely specified model (option 1, most conservative) to not providing any prior information (option 4, least conservative). We investigated tissue homogeneity on a voxel-by-voxel basis, and while some deviations must be expected, a stronger deviation within a given approach is indicative of a less-homogeneous tissue map. As expected (Table 2), the more conservative approaches result in more homogeneous tissue maps, with the lack of a starting estimate (option 4) resulting in the most inhomogeneous maps. To address this, we used a *post-hoc* 3D median filtering. The default (small) amount of filtering applied further improved quality, but could conceivably be explored further to also include robust (Garcia, 2010) or Markov Random field filtering approaches (Rajapakse et al., 1997); however, neither substantially improved image quality in preliminary testing rounds, and was thus not explored further.

Homogeneity of the resulting maps, however, is only one point arguing in favor of one or the other approach. The variance explained by the fitted model was therefore also assessed on a voxel-wise basis, using functionality available in the ARESlab toolbox (for the spline-based approaches; Jekabsons, 2011) or Matlab (for the GLM approach). When assessing this over all approaches in the three primary tissue classes, there is a clear trend of more variance being explained by the less conservative approaches. As these are less-constrained, this is as expected, but the overall absolute effect is low. Of note, the previously-suggested GLM approach (Wilke et al., 2008) only outperforms the spline-based option 1, despite having more regressors (age ^2^ and age ^3^) in the model. In every other scenario, the newer approach outperforms the previously-suggested one. Considering the arguments pertaining to biological plausibility as well as the results presented in Tables 2, 3, option 2 seems the currently best compromise between flexibility and stringency.

### Resulting tissue priors and possible extensions

One aim of this study was to explore the applicability of a multivariate adaptive regression splines approach for template creation. The resulting six-class tissue maps can be used as targets for spatial normalization as well as the basis for tissue segmentation in current segmentation approaches (Ashburner, 2012; Gaser, 2014, Malone et al., 2015), providing more appropriate targets for “unusual” populations in particular. All resulting maps as shown in Figure 4 are of high quality, and, depending on the processing approach and in line with the results shown in Figures 2, 3, show more or less effects of age.

When exploring the effects of the different covariates, it is interesting that field strength still has a relevant, and systematic, impact on the resulting tissue maps (Figure 5), despite our attempts to alleviate the influence by additional pre-processing steps. This confirms field strength to be a relevant factor for tissue segmentation, on the individual (Marques et al., 2010; West et al., 2013) as well as on the group level (Wilke et al., 2014). The fact that the effect of gender is not as prominent may seem surprising but is actually well in line with the observation that its impact is on the global, rather than on the regional level (Brain Development Cooperative Group, 2012; Wilke et al., 2014). These are already accounted for by the affine transformation; the remaining effect in the processed (density) images is therefore not as prominent anymore. Finally, the more scattered, but quite obvious effects of image quality is interesting and underlines the detrimental effect of image quality on tissue segmentation (Camara-Rey et al., 2006; Preboske et al., 2006, Shuter et al., 2008). Taken together, the quite distinct contributions of these different variables are another point arguing in favor of explicitly identifying and removing such effects when generating tissue priors, instead of blindly including them (in the conventional approach to template creation).

When simulating conventionally generated tissue maps for three scenarios (with 25, 50, or 100 subjects), the corresponding CerebroMatic tissue maps are closer to the gold standard (the overall population mean) for gray (Figure 6) and white matter maps (Figure 7). This demonstrates that the correspondence with the gold standard was substantially greater for the synthetic priors, for smaller groups in particular. It is also interesting to notice that the variance within each group (of 100 simulations), as well as between the scenarios (of 25/50/100 subjects), is much lower for the CerebroMatic priors, suggesting that more consistently reproducible maps were obtained when using a statistical modeling approach.

In further simulations (Figure 8), the correspondence between the synthetically-generated maps and the overall population mean continues to increase with group size, until the maximum (*n* = 943) is reached for gray matter. For white matter, a plateau seems to be reached at about 800 subjects, suggesting that no further increase in quality is achieved when adding more subjects for this tissue class. It must be remembered that the CerebroMatic tissue maps were always matched to 25 random subjects from the respective population, resulting in maps with a very low variance (cf. Figures 6, 7). Hence, the ever-increasing correspondence is solely due to the increasing size of the control population. The fact that the mean of more subjects (leading to tissue maps of increasingly higher quality) shows a lower deviation from the synthetically generated maps strongly argues for these latter maps to be of a very high standard.

As demonstrated here and previously (Wilke et al., 2002), the amount of deformation is substantially influenced by the “distance” of the individual subject to the tissue prior used for normalization. The benefits of using a less-deformed image (preserving more of the original variability) must be weighed against the disadvantage of obtaining less sharply-defined output images (such as shown in Figure 4, middle row). Instead of taking these as the final priors, however, they could be subjected to a within-group-registration approach such as DARTEL (Ashburner, 2007) or the more recently-developed SHOOT algorithm (Ashburner and Friston, 2011; SPM12, 2015). The advantage of using such an approach on the generated priors is that the age-related variance has already been accounted for in the initial modeling step. For example, matching priors for 15 subjects could be submitted to such a groupwise iterative registration approach, potentially generating tissue priors that are more representative in both shape and intensity (Ashburner and Friston, 2009). This has not worked for our previous data (Wilke et al., 2008) as the resulting images were too smooth, giving the registration approaches no intensity gradients to work on. In preliminary testing, this approach was shown to generate more crisp priors from the unified segmentation approach, and could thus be used as an adjunct.

### Possible limitations

It should be remembered that the Jacobian determinant is not a fully representative indicator of “deformation,” broadly defined: a simple displacement of a given voxel will not change its volume. Also, the Jacobian itself is influenced by how the non-linear deformation is regularized (see Ashburner and Ridgway, 2013, for a discussion). However, it is an intuitive and well-established measure of volume change, and widely-used in imaging neuroscience and beyond; we therefore also decided to use it here.

When removing datasets from a population as part of a quality control step, the criteria for “insufficient quality” must clearly be established beforehand, to prevent that the final sample does not fully reflect the original population (an issue known as “tyding up” bias; Sackett, 1979). On the other hand, the impact of image non-idealities on the result of VBM-type studies may be substantial (Camara-Rey et al., 2006; Preboske et al., 2006; Shuter et al., 2008; see also Figure 5). With our stringently implemented, *a priori* defined procedure rejecting only ~8% of subjects, we believe our approach to be defendable.

Achieving an accurate overlap of cortical structures has been recognized to be difficult for pure volume-based approaches, and the use of additional surface-based matching approaches was suggested (Luo et al., 2014), for the use in developing populations in particular (Ghosh et al., 2010). However, there is no universal advantage of the one vs. the other approach (Klein et al., 2010), and intensity/volume-based approaches continue to be in widespread use. Also, approaches such as the above-mentioned extension might be able to achieve a similar goal, we therefore do not see this as a major limitation. Further, registration based on cortical connectivity patterns (Zhu et al., 2013) or white matter microstructure (Varentsova et al., 2014) has been suggested. While no comparison between approaches was attempted here, it seems relevant to notice that the approach suggested here may also aid in the generation of target brains based on other modalities, or even species, as no assumption is made about the underlying data and meaningful trends inherent in the data can appropriately be described.

The definition of defaults as described above was based on biological considerations and constraints, but was not exhaustively tested on the dataset at hand. Further analyses regarding the trends inherent in the data, and more formal model selection criteria such as the Akaike or the Bayesian information criteria (Stoica and Selen, 2004) could be used in future simulations to find the best-possible combination of parameters for modeling. Also, other factors such as race (Tang et al., 2010) could be included as demographic variables, allowing to explain even more unwanted variance during model estimation. Also, other approaches to enforce homogeneity could be tested, such as a hierarchical modeling (first on an intermediate, then on the voxel level) approach. However, this is computationally demanding: the generation of the initial statistical parameters takes several days on a high-performance computational cluster, and resulting files (especially for the background class, with many non-zero voxels) are rather large. However, while being memory intensive, the ultimate generation of the tissue priors is a matter of minutes on a current workstation, and as the parameters can be made available, this will be the only step relevant to future users of this approach.

Finally, it must be stated that the effect of using spline-based vs. conventionally-generated templates has not been investigated here. In the absence of a ground truth, such effects would be difficult to assess, and it was decided that this would be beyond the scope of this current technical study. It was also not evaluated in how far such statistical representations of “normal” would be helpful in the automated detection of local brain abnormalities (Wilke et al., 2014).

## Conclusions

We here present a novel approach to template creation that allows for the generation of tissue class priors from infancy to old age, and make the resulting CerebroMatic toolbox employing this approach available to the neuroscience community. Their generation is conceptually sound, versatile, and yields tissue priors of reproducibly high quality that are closer to the gold standard than conventionally-generated tissue maps. The potentially resulting increase in cross-study variance (due to a larger number or templates) may therefore be offset by an increase in detection power within each study, due to a more appropriate data processing approach particularly for “unusual” populations such as infants, young children, and elderly subjects.

## Author contributions

This work was conceived and developed in joint discussions (MW, MA, SH) and was advanced by processing the data (MW) and generating the results and the figures (MW). The interpretation of results and the identification of major points to discuss were again achieved together (MW, MA, SH), with each scientist contributing specific MR image data processing (MW), methodological (MW, SH) or statistical (MA) scientific expertise. SH and MA contributed as part of their commitment to the Cincinnati MR Imaging of Neurodevelopment (CMIND) study; this authorship consortium is therefore also listed.

## Funding

This work was partly performed on the computational resource bwUniCluster funded by the Ministry of Science, Research and Arts and the Universities of the State of Baden-Württemberg, Germany, within the framework program bwHPC. This works was sponsored by the Eunice Kennedy Shriver National Institute of Child Health and Human Development (NICHD) Contract title: The Pediatric Functional Neuroimaging Research Network, #HHSN275200900018C. This study was also funded in part by a grant from the H.W. and J. Hector Foundation, Mannheim (M66, to MW). Neither sponsor had any role in study design, in the collection, analysis and interpretation of data, in the writing of the report, and in the decision to submit the article for publication.

### Conflict of interest statement

The authors declare that the research was conducted in the absence of any commercial or financial relationships that could be construed as a potential conflict of interest.
