# Supplementary material for: CerebroMatic: A Versatile Toolbox for Spline-Based MRI Template Creation
Source: Front Comput Neurosci. 2017 Feb 22;11:5. doi: 10.3389/fncom.2017.00005 (PMC5321046; doi:10.3389/fncom.2017.00005)
Supplement: Supplementary file 1 [file DataSheet1.PDF]

CerebroMatic: a versatile toolbox for spline-based MRI template creation

Marko Wilke [1], Mekibib Altaye [2], Scott K. Holland [3], and the CMIND authorship consortium

[1] *Department of Pediatric Neurology and Developmental Medicine, Children's Hospital* and *Experimental Pediatric Neuroimaging group, Children's Hospital & Dept. of Neuroradiology, University of Tübingen, Germany*

[2] *Pediatric Neuroimaging Research Consortium, Cincinnati Children's Research Foundation* and *Department of Pediatrics, Division of Biostatistics and Epidemiology, University of Cincinnati College of Medicine, Cincinnati, OH, USA*

[3] *Pediatric Neuroimaging Research Consortium, Cincinnati Children's Research Foundation* and *Department of Radiology, University of Cincinnati College of Medicine, Cincinnati, OH, USA*

Supplementary Material S1

Supplementary Figure 1: overview of the data processing and analysis pipeline

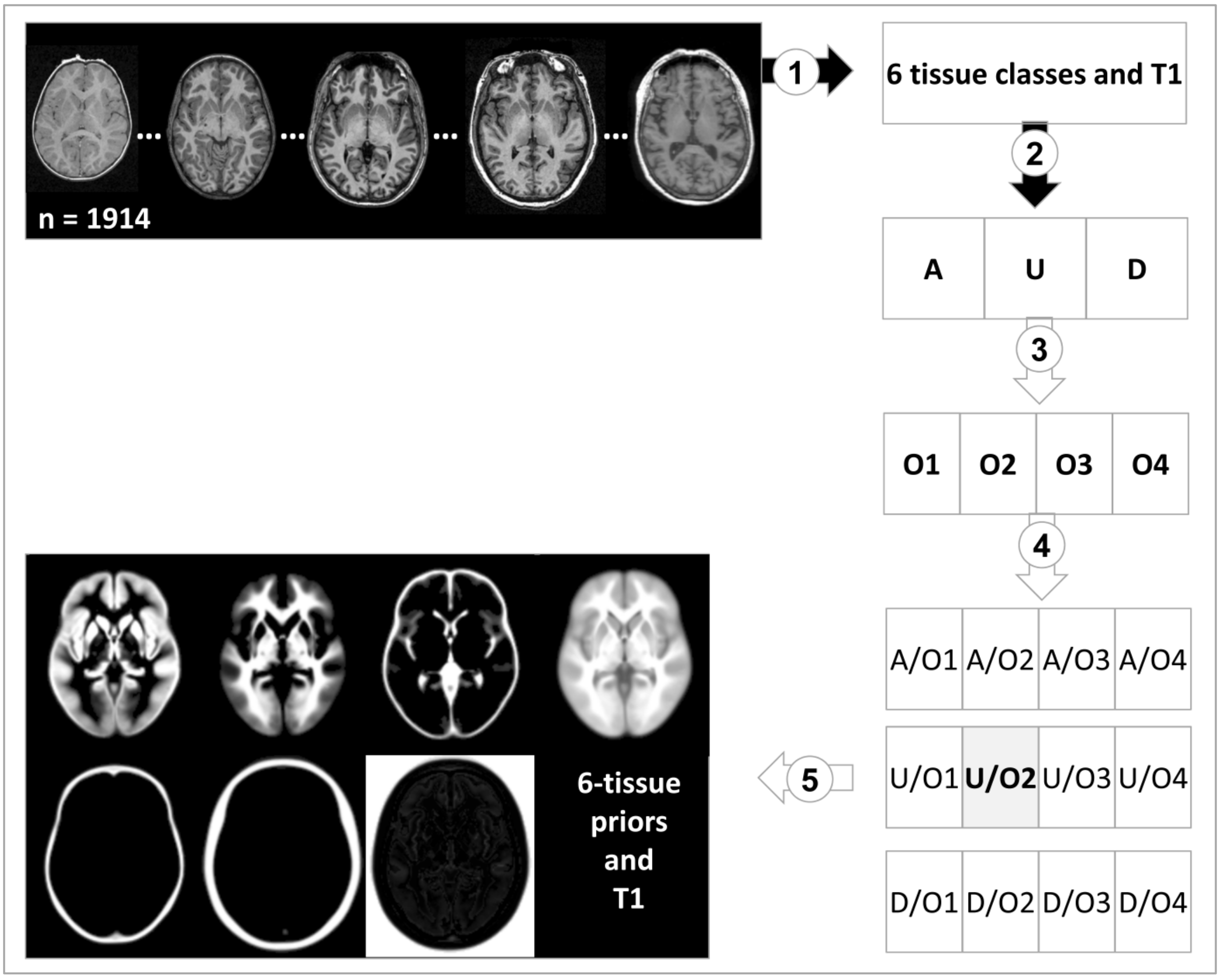

The complete dataset of 1914 images (covering the age range from 13 months to 75 years) is segmented into 6 tissue classes [step 1], applying three different spatial normalization approaches: affine only (A), unified segmentation (U), and DARTEL (D) [step 2]. In the multivariate adaptive regression spline analysis stage, four data processing options are evaluated (O1-O4) [step 3], resulting in a total of 12 datasets which were compared with regard to their performance [step 4]. Following the identification of the best-performing approach (U/O2, shaded), the statistical parameters determined at each voxel can be used to generate a 6-class tissue prior and a matching T1 dataset [step 5].

Supplementary material: CMIND authorship consortium names and affiliations

The Cincinnati MR Imaging of NeuroDevelopment (CMIND) authors are:

Scott K. Holland, Ph.D. [1,6,9,10]
Jennifer Vannest, Ph.D. [1,5]
Vincent J. Schmithorst, Ph.D. [1,2]
Mekibib Altaye, Ph.D. [1,7]
Gregory Lee, Ph.D. [1,6]
Luis Hernandez-Garcia, Ph.D. [3]
Michael Wagner, Ph.D. [1,8]
Arthur Toga, Ph.D. [12,13]
Jennifer Levitt, MD [14]
Anna W. Byars, Ph.D. [1,5]
Andrew Dimitrijevic, Ph.D.[9,10]
Nicolas Felicelli [8]
Darren Kadis, Ph.D. [1,5]
James Leach, MD [1,6]
Katrina Peariso, MD, Ph.D. [5]
Elena Plante, Ph.D. [4]
Akila Rajagopal, M.S. [1]
Andrew Rupert, M.S. [8]
Mark Schapiro, MD [1,5]
Karen Crawford [12]
Ronald Ly [14]
Katherine Narr, Ph.D. [11]
Petros Petrosyan [12]
JJ Wang, Ph.D. [11]
Lisa Freund, Ph.D. [15]

[1] Pediatric Neuroimaging Research Consortium
[5] Div. of Neurology, Dept. of Pediatrics
[6] Dept. of Radiology
[7] Div. of Biostatistics and Epidemiology, Dept. of Pediatrics
[8] Div. of Biomedical Informatics, Dept. of Pediatrics
[9] Dept. of Otolaryngology
[10] Communication Sciences Research Center
Cincinnati Children's Hospital Medical Center, University of Cincinnati

[2] Pediatric Imaging Research Center, Dept. of Radiology
Children's Hospital of Pittsburgh of UPMC, Pittsburgh, PA

[3] Functional MRI Laboratory
Department of Biomedical Engineering, University of Michigan, Ann Arbor, MI

[4] Dept. of Speech, Language, and Hearing Sciences
University of Arizona, Tucson, AZ

[11] Dept. of Neurology, UCLA
[12] Laboratory of Neuroimaging, Keck School of Medicine of USC
[13] Departments of Ophthalmology, Neurology, Psychiatry, and the Behavioral Sciences, Radiology and Engineering, Keck School of Medicine of USC
[14] Psychiatry and Biobehavioral Sciences, UCLA
Los Angeles, CA

[15] Eunice Kennedy Shriver National Institute of Child Health and Human Development
Bethesda, MD

Supplementary material: detailed listing of subject IDs from all datasets

Dataset 1: From the *NIH Study of normal brain development* (NIH, Bethesda, MD, USA), URL: http://pediatricmri.nih.gov/nihpd/info/index.html, subjects with the following IDs were used: 1001, 1002, 1003, 1004, 1005, 1007, 1008, 1009, 1010, 1011, 1012, 1013, 1014, 1015, 1017, 1018, 1019, 1020, 1021, 1023, 1024, 1025, 1026, 1027, 1028, 1029, 1030, 1031, 1032, 1033, 1034, 1035, 1036, 1037, 1038, 1039, 1040, 1041, 1042, 1043, 1044, 1045, 1046, 1047, 1051, 1052, 1053, 1055, 1056, 1057, 1058, 1059, 1061, 1062, 1063, 1064, 1065, 1066, 1067, 1068, 1069, 1070, 1071, 1073, 1074, 1075, 1076, 1077, 1078, 1079, 1080, 1081, 1082, 1083, 1084, 1085, 1086, 1087, 1088, 1089, 1090, 1091, 1092, 1093, 1094, 1095, 1096, 1097, 1098, 1099, 1100, 1102, 1104, 1105, 1106, 1107, 1108, 1109, 1110, 1111, 1112, 1113, 1114, 1116, 1118, 1119, 1121, 1122, 1123, 1124, 1125, 1126, 1127, 1128, 1129, 1130, 1131, 1132, 1133, 1134, 1136, 1138, 1139, 1140, 1141, 1142, 1143, 1144, 1145, 1146, 1147, 1148, 1149, 1150, 1153, 1154, 1155, 1157, 1158, 1159, 1160, 1161, 1162, 1163, 1164, 1165, 1166, 1167, 1168, 1169, 1170, 1171, 1172, 1173, 1174, 1175, 1176, 1177, 1178, 1179, 1180, 1181, 1182, 1183, 1184, 1186, 1187, 1188, 1189, 1190, 1191, 1192, 1193, 1194, 1195, 1196, 1197, 1199, 1200, 1201, 1202, 1203, 1205, 1206, 1207, 1208, 1209, 1210, 1211, 1213, 1215, 1216, 1217, 1218, 1219, 1220, 1221, 1222, 1223, 1224, 1225, 1226, 1227, 1228, 1229, 1230, 1231, 1232, 1233, 1234, 1235, 1236, 1237, 1238, 1239, 1240, 1241, 1242, 1244, 1245, 1246, 1247, 1248, 1249, 1251, 1252, 1253, 1254, 1255, 1257, 1258, 1259, 1260, 1262, 1263, 1264, 1265, 1266, 1267, 1268, 1269, 1270, 1271, 1272, 1273, 1274, 1275, 1276, 1277, 1278, 1280, 1281, 1282, 1284, 1285, 1287, 1288, 1289, 1290, 1291, 1292, 1294, 1297, 1298, 1299, 1300, 1301, 1302, 1303, 1304, 1305, 1306, 1307, 1308, 1309, 1310, 1311, 1312, 1313, 1314, 1315, 1316, 1317, 1318, 1319, 1320, 1321, 1322, 1323, 1324, 1325, 1326, 1328, 1329, 1330, 1331, 1332, 1333, 1334,

1335, 1336, 1337, 1338, 1339, 1340, 1341, 1342, 1343, 1344, 1346, 1348, 1349, 1350, 1351, 1352, 1353, 1354, 1356, 1357, 1358, 1359, 1360, 1361, 1362, 1363, 1364, 1365, 1367, 1368, 1369, 1370, 1371, 1372, 1373, 1374, 1376, 1377, 1379, 1380, 1382, 1383, 1384, 1386, 1387, 1388, 1389, 1390, 1391, 1392, 1396, 1397, 1398, 1399, 1400, 1401, 1402, 1403, 1404, 1405, 1406, 1407, 1408, 1409, 1410, 1411, 1413, 1414, 1415, 1416, 1417, 1418, 1419, 1420, 1422, 1423, 1424, 1425, 1426, 1427, 1428, 1429, 1430, 1431, 1432, 1441, 1444, 1448, 1471, 1521

Please note: this manuscript reflects the views of the author and may not reflect the opinions or views of the Brain Development Cooperative Group Investigators or the NIH. The contract numbers for the NIH MRI study of normal brain development were N01-HD02-3343, N01-MH9-0002, and N01-NS-9-2314, 2315, 2316, 2317, 2319 and 2320. A listing of the participating sites and a complete listing of the study investigators can be found at the website of the data coordinating center at www.bic.mni.mcgill.ca/nihpd/info/participating_centers.html.

Dataset 2: From Cincinnati MR Imaging of NeuroDevelopment (C-MIND), URL: https://research.cchmc.org/c-mind/, subjects with the following IDs were used: 00M034_C, 01F011_C, 01F014_C, 01F015_C, 01F020_C, 01F032_C, 01F033_C, 01F036_C, 01F037_C, 01F043_C, 01F044_C, 01M001_C, 01M002_U, 01M005_C, 01M008_C, 01M029_C, 01M035_C, 02F003_C, 02F011_C, 02F016_C, 02F020_C, 02F021_C, 02F022_C, 02F023_C, 02F028_C, 02F029_C, 02F031_C, 02F035_C, 02F043_C, 02F044_C, 02M015_C, 02M018_C, 02M021_C, 02M022_C, 02M034_C, 03F001_C, 03F003_C, 03F011_C, 03F017_C, 03F023_C, 03F025_C, 03F026_C, 03F033_C, 03F034_C, 03M001_C, 03M005_C, 03M011_C, 03M017_C, 03M019_C, 03M033_C, 03M034_C, 03M035_C, 04F001_C, 04F018_C, 04F019_C, 04F022_C, 04M005_C, 05F002_C, 05F003_C, 05F006_C, 05F012_C, 05F015_C, 05F016_C, 05F017_C, 05M001_C, 05M002_C, 05M003_C, 05M004_C, 06F004_C, 06F010_C, 06F014_C, 06F015_C, 06F016_C, 06M002_C, 06M007_C, 06M008_C, 06M011_C, 06M014_C, 06M018_C, 07F001_C, 07F002_C, 07F002_U, 07F003_C, 07F003_U, 07F004_C, 07F010_C, 07F012_C, 07M002_U, 07M003_C, 07M005_C, 07M005_U, 07M006_U, 07M007_U, 07M008_C, 07M008_U, 07M009_C, 07M010_C, 07M014_C, 07M017_C, 08F002_U, 08F003_C, 08F004_C, 08F005_C, 08F005_U, 08F006_C, 08F006_U, 08F007_U, 08F008_C, 08F008_U, 08F009_U, 08F010_C, 08F010_U, 08F013_C, 08F014_C, 08F015_C, 08F016_C, 08F017_C, 08F019_C, 08F020_C, 08F021_C, 08M002_C, 08M002_U, 08M003_U, 08M004_U, 08M008_C, 08M010_C, 08M011_C, 08M012_C, 08M017_C, 09F002_C, 09F002_U, 09F004_U, 09F005_U, 09F006_U, 09F007_U, 09F009_C, 09F009_U, 09F010_U, 09F012_C, 09F017_C, 09M001_U, 09M002_U, 09M003_U, 09M004_C, 09M004_U, 09M005_C, 09M005_U, 09M007_C, 09M013_C, 09M014_C, 09M021_C, 10F001_C, 10F002_C, 10F005_C, 10F006_C, 10M002_C, 10M004_C, 11F004_C, 11F007_C, 11F008_C, 11M001_C, 11M002_C, 11M005_C, 11M007_C, 12F001_C, 12F004_C, 12F005_C, 12F007_C, 12M002_C,

12M003_C, 12M007_C, 12M008_C, 13F003_C, 13F004_C, 13F005_C, 13F007_C, 13M001_C, 13M003_C, 13M004_C, 13M015_C, 13M018_C, 14F001_C, 14F002_C, 14F003_C, 14F006_C, 14F009_C, 14M006_C, 14M012_C, 14M014_C, 15F001_C, 15F002_C, 15M005_C, 15M013_C, 15M015_C, 15M022_C, 16F005_C, 16F007_C, 16M002_C, 16M007_C, 16M010_C, 16M014_C, 17F002_C, 17F004_C, 17M003_C, 17M004_C, 17M010_C, 18F002_C, 18F004_C, 18F011_C, 18F012_C, 18M001_C

Please note: data presented in this work was obtained from the database known as Cincinnati MR Imaging of NeuroDevelopment (C-MIND), provided by the Pediatric Functional Neuroimaging Research Network at https://research.cchmc.org/c-mind/. This Network and the resulting C-MIND database was supported by contract from the Eunice Kennedy Shriver National Institute of Child Health and Human Development (HHSN275200900018C).

Dataset 3: From The 1000 Functional Connectomes Project, URL: http://www.nitrc.org/ir/app/action/ProjectDownloadAction/project/fcon_1000, subjects with the following IDs were used: from Ann Arbor : 00306, 04111, 04619, 07921, 11043, 13636, 13959, 15846, 16960, 18546, 18698, 20317, 26099, 28433, 30250, 30421, 33437, 34781, 38614, 39635, 39923, 42616, 43409, 45569, 45660, 46727, 47191, 47659, 49687, 51248, 53269, 53959, 56028, 56686, 57025, 59573, 62819, 64831, 64969, 70106, 72215, 73812, 75922, 78151, 82334, 85257, 86367, 90950, 96621, 97518, 98007, 99692; from Atlanta: 00354, 00368, 06870, 07145, 15817, 18219, 18702, 24972, 26938, 32093, 49816, 53122, 55652, 58250, 59806, 60499, 61442, 61902, 71337, 72096, 72971, 75153, 76280, 81596, 86323, 91049; from Baltimore: 17017, 19738, 23750, 23927, 29158, 30072, 31837, 37548, 52358, 54257, 54329, 73823, 76160, 77572, 80221, 81887, 85922, 86414, 90658, 90893, 91622, 94042, 96234; from Bangor: 00031, 01903, 03557, 04097, 14388, 27519, 36736, 46870, 48632, 61418, 61908, 63767, 66585, 68050, 73082, 77520, 81464, 82625, 87568, 91556; from Beijing: 00440, 01018, 01244, 02403, 04050, 04191, 05267, 06880, 06899, 07144, 07716, 07717, 08001, 08251, 08455, 08816, 08992, 10186, 10277, 10869, 10973, 11072, 11344, 12220, 14238, 15441, 16091, 16943, 17093, 17159, 17315, 17586, 17603, 17642, 17688, 18326, 18758, 18960, 19642, 19974, 20127, 20246, 20765, 20948, 21115, 22201, 22595, 22661, 22715, 22890, 26713, 28206, 28403, 28698, 28792, 28801, 28907, 28965, 29590, 29785, 30272, 30310, 30556, 30616, 30988, 31058, 31729, 32517, 32587, 33747, 33943, 33991, 34895, 34943, 35309, 35776, 35806, 36580, 36942, 37602, 38602, 39725, 40037, 40427, 41170, 41621, 42512, 42555, 42843, 43290, 44573, 45552, 46058, 46259, 46541, 48501, 48563, 48676, 49782, 50498, 50873, 50972, 50985, 51015, 51586, 52044, 52259, 53572, 53998, 54890, 55301, 55541, 55736, 55856, 56136, 56659, 56703, 56757, 58029, 58332, 58614, 59347, 59448, 61961, 62083, 62438, 62843,

62966, 64923, 65467, 65659, 66158, 66528, 66781, 66889, 67435, 67844, 68012, 68597, 69518, 69696, 71693, 72654, 72678, 73098, 73245, 73279, 73421, 74386, 74587, 75878, 76377, 77440, 80163, 80551, 80569, 80927, 81062, 81074, 82352, 82426, 82714, 82826, 82980, 83430, 83624, 83728, 85030, 85543, 85818, 86114, 87089, 87776, 88306, 88947, 89088, 89238, 89592, 89742, 89941, 91145, 91399, 91952, 92430, 92490, 92544, 92602, 92799, 92859, 93689, 93856, 94536, 95575, 95755, 96163, 97442, 98353, 98617; from Berlin: 06204, 06716, 12855, 18913, 23506, 27536, 27711, 27797, 28092, 33248, 38279, 40143, 47066, 47791, 49134, 54976, 57028, 67166, 75506, 77281, 85681, 86111, 91116, 91966, 95068, 97162; from Cambridge: 00156, 00294, 01361, 02591, 02953, 04187, 04270, 04491, 04665, 05306, 05453, 06037, 06272, 06987, 07413, 07798, 07902, 08204, 08588, 08723, 08947, 09015, 09397, 09633, 10268, 10619, 11388, 12346, 13093, 13187, 13216, 13902, 14183, 14194, 14278, 15172, 15258, 15432, 15905, 16122, 16390, 16846, 17584, 17737, 17772, 18295, 18449, 19717, 20389, 20543, 20563, 21755, 23780, 23869, 24670, 24757, 25044, 25058, 25099, 26348, 27065, 27230, 27613, 27796, 29044, 29425, 29800, 31522, 34586, 34741, 35430, 35512, 37374, 39053, 39065, 39142, 39737, 40635, 41567, 41773, 41814, 42146, 42253, 43304, 43358, 45344, 45354, 45604, 47162, 47231, 47278, 47498, 49259, 49998, 50272, 50454, 50953, 51050, 51172, 51512, 51671, 52036, 52300, 52442, 53059, 53107, 53193, 53296, 53615, 54846, 55114, 55660, 55874, 57221, 58360, 58470, 58682, 58874, 59434, 59729, 60578, 60797, 61185, 61209, 61436, 61753, 62383, 62424, 62732, 62908, 63412, 63661, 64308, 64985, 65373, 65682, 66351, 67117, 68101, 68425, 69287, 69315, 69397, 71849, 72068, 73317, 73399, 73477, 76631, 76745, 77337, 77435, 77598, 77989, 78547, 78552, 78614, 80557, 81289, 81524, 81562, 82113, 82213, 82435, 83409, 83683, 84064, 84256, 84504, 84845, 86115, 86637, 87846, 88445, 88464, 88853, 89107, 89435, 89894, 90059, 90674, 90681, 90699, 92288, 92393, 92440, 93269,

93488, 93609, 94304, 95187, 95644, 95959, 98528, 98624, 99085, 99330, 99462; from Cleveland: 02480, 07835, 12330, 13495, 17946, 18011, 18566, 19005, 20003, 22736, 22935, 26557, 28596, 34189, 46075, 46739, 47482, 50092, 58811, 61868, 64706, 65858, 67936, 75398, 76139, 80263, 82518, 85091, 92232, 97844, 99664; from ICBM: 00448 , 05208 , 10582 , 16607 , 26183 , 28808 , 32549 , 35262 , 40217 , 44077 , 49215 , 55114 , 59914 , 68850 , 77431 , 85442 , 94945 , 98802 , from Leiden: 01553, 01787, 04484, 08518, 09796, 10481, 12255, 13537, 18456, 19281, 28473, 30943, 36743, 38454, 39335, 40907, 52853, 52922, 56299, 57187, 58194, 64642, 66131, 68050, 72247, 86034, 87320, 92061, 93194, 97690, 99856; from Milwaukee: 00917, 09931, 14692, 16666, 17004, 17987, 18955, 21350, 23607, 24237, 28782, 30157, 36386, 39259, 44912, 45019, 45852, 46312, 49975, 50771, 51182, 53971, 55176, 56084, 56108, 56333, 56582, 58677, 58967, 59359, 61779, 63196, 64463, 67948, 73547, 75919, 76042, 76378, 77073, 84314, 87784, 87910, 91468, 93170, 98971, 99479; from Munchen: 09035, 26223, 26670, 28697, 28902, 31272, 36052, 50162, 64202, 66933, 70942, 72095, 74607, 81737, 96591, 96752, from Newark: 32580, 36023, 41006, 43517, 49705, 53422, 54933, 55760, 58526, 59397, 59799, 62985, 71042, 71743, 76647, 78225, 86204; from New York: 01912, 02503, 03951, 04856, 05208, 07578, 08595, 09539, 10011, 10582, 12486, 13384, 14299, 14465, 15213, 15758, 16607, 17078, 17109, 18638, 19579, 20676, 20691, 20732, 21212, 22349, 22608, 23844, 24528, 26267, 27123, 28795, 28808, 29216, 29353, 29935, 30247, 30623, 30860, 31554, 33062, 33581, 35262, 37864, 38088, 41546, 44395, 44979, 45217, 46856, 47087, 47633, 48803, 48830, 50559, 51309, 51677, 53461, 53710, 54828, 56734, 59796, 63915, 69779, 73035, 77203, 77903, 84371.

Dataset 4: From the IXI-Dataset, Biomedical Image Analysis Group (Imperial College, London, UK), URL: http://biomedic.doc.ic.ac.uk/brain-development/index.php?n=Main.Datasets, subjects with the following IDs were used: 002, 012, 013, 014, 015, 016, 017, 019, 020, 021, 022, 023, 024, 025, 026, 027, 028, 029, 030, 031, 033, 034, 035, 036, 037, 038, 039, 040, 041, 042, 043, 044, 045, 046, 048, 049, 050, 051, 052, 053, 054, 055, 056, 057, 058, 059, 060, 061, 062, 063, 064, 065, 066, 067, 068, 069, 070, 071, 072, 073, 074, 075, 076, 077, 078, 079, 080, 083, 084, 085, 086, 087, 089, 090, 091, 092, 093, 094, 095, 096, 097, 098, 099, 100, 101, 102, 103, 104, 105, 106, 107, 108, 109, 110, 111, 112, 113, 114, 115, 116, 118, 119, 120, 121, 122, 123, 126, 127, 128, 129, 130, 131, 132, 134, 135, 136, 137, 138, 139, 140, 141, 142, 143, 144, 145, 146, 148, 150, 151, 153, 154, 156, 157, 159, 160, 161, 162, 163, 164, 165, 166, 167, 168, 169, 170, 172, 173, 174, 175, 176, 177, 178, 179, 180, 181, 182, 183, 184, 186, 188, 189, 191, 192, 193, 194, 195, 196, 197, 198, 199, 200, 201, 202, 204, 205, 206, 207, 208, 209, 210, 211, 212, 213, 214, 216, 217, 218, 219, 221, 222, 223, 224, 225, 226, 227, 229, 230, 231, 232, 233, 234, 236, 238, 239, 240, 241, 242, 244, 246, 247, 248, 249, 250, 252, 253, 254, 255, 256, 257, 258, 259, 260, 261, 262, 263, 264, 265, 266, 267, 268, 269, 270, 274, 275, 276, 277, 278, 279, 280, 282, 284, 285, 286, 287, 289, 290, 291, 292, 293, 294, 295, 296, 297, 298, 299, 300, 302, 303, 304, 305, 306, 307, 308, 309, 310, 311, 312, 313, 314, 315, 316, 317, 318, 319, 320, 321, 322, 324, 325, 326, 327, 328, 329, 330, 331, 332, 334, 335, 336, 338, 342, 344, 348, 350, 351, 353, 354, 356, 357, 358, 359, 360, 361, 362, 363, 364, 365, 367, 368, 369, 370, 371, 372, 373, 375, 376, 377, 378, 379, 380, 381, 382, 383, 384, 385, 386, 387, 388, 389, 390, 391, 392, 393, 394, 395, 396, 397, 398, 399, 400, 401, 402, 403, 404, 405, 406, 407, 408, 409, 410, 411, 412, 413, 414, 415, 416, 417, 418, 419, 420, 422, 423, 424, 425, 426, 427, 428, 429, 430, 431, 432, 433, 434, 436, 437, 438, 439, 440, 441, 442, 443, 444, 445, 446, 447, 449, 450, 451, 452, 453, 454, 455, 456, 458, 459, 460, 461, 463, 465, 467, 468, 469, 470, 473, 474, 475, 477, 478, 479,

480, 481, 482, 483, 484, 485, 486, 487, 488, 489, 490, 492, 493, 494, 495, 496, 497, 500, 501, 502, 503, 504, 505, 506, 507, 508, 510, 511, 512, 515, 516, 517, 519, 521, 522, 523, 524, 525, 526, 527, 528, 532, 533, 534, 535, 536, 537, 541, 542, 543, 544, 546, 547, 548, 549, 550, 551, 552, 553, 554, 555, 556, 558, 559, 560, 561, 562, 563, 565, 566, 567, 568, 569, 571, 572, 573, 574, 575, 576, 577, 578, 579, 582, 584, 585, 586, 587, 588, 591, 592, 593, 594, 595, 596, 597, 598, 599, 600, 601, 603, 605, 606, 608, 609, 610, 611, 612, 613, 614, 616, 617, 618, 619, 621, 622, 625, 626, 627, 629, 630, 631, 632, 633, 634, 635, 636, 641, 642, 644, 646, 648, 651, 652, 653, 662.
